# Impact of multidomain preventive strategies on functional brain connectivity in older adults with cognitive complaint: Subset from the Montpellier center of the ancillary MAPT-MRI study

**DOI:** 10.3389/fnagi.2022.971220

**Published:** 2023-01-10

**Authors:** Lisa Perus, Jean-François Mangin, Jérémy Deverdun, Laure-Anne Gutierrez, Emmanuelle Gourieux, Clara Fischer, Liesjet E. H. Van Dokkum, Clara Manesco, Germain Busto, Sophie Guyonnet, Bruno Vellas, Audrey Gabelle, Emmanuelle Le Bars, The MAPT/DSA group, Bruno Vellas

**Affiliations:** ^1^Memory Resources and Research Center, Department of Neurology, Gui de Chauliac Hospital, Montpellier, France; ^2^INM, Univ Montpellier, INSERM, CHU Montpellier, Montpellier, France; ^3^Institut d'Imagerie Fonctionnelle Humaine, I2FH, Department of Neuroradiology, Gui de Chauliac Hospital and University of Montpellier, Montpellier, France; ^4^CATI, US52-UAR2031, CEA, ICM, SU, CNRS, INSERM, APHP, Ile de France, France; ^5^Université Paris-Saclay, CEA, CNRS, Neurospin, UMR9027 Baobab, Gif-sur-Yvette, France; ^6^Laboratoire Charles Coulomb (L2C), University of Montpellier, CNRS, Montpellier, France; ^7^Inserm UMR 1295, University of Toulouse III, Toulouse, France; ^8^Gérontopôle, Department of Geriatrics, CHU Toulouse, Toulouse, France

**Keywords:** magnetic resonance imaging (MRI), resting-state functional MRI (rs-fMRI), multidomain intervention, exercise, cognitive training, omega-3 fatty acids

## Abstract

**Introduction:**

The impact of multi-domain preventive interventions on older adults, in particular on those with higher risk to develop Alzheimer's disease (AD), could be beneficial, as it may delay cognitive decline. However, the precise mechanism of such positive impact is not fully understood and may involve brain reserve and adaptability of brain functional connectivity (FC).

**Methods:**

To determine the effect of multidomain interventions (involving physical activity, cognitive training, nutritional counseling alone or in combination with omega-3 fatty acid supplementation and vs. a placebo) on the brain, longitudinal FC changes were assessed after 36 months of intervention on 100 older adults (above 70 year-old) with subjective cognitive complaints.

**Results:**

No global change in FC was detected after uni or multidomain preventive interventions. However, an effect of omega-3 fatty acid supplementation dependent on cognitive decline status was underlined for frontoparietal, salience, visual and sensorimotor networks FC. These findings were independent of the cortical thickness and vascular burden.

**Discussion:**

These results emphasize the importance of patient stratification, based on risk factors, for preventive interventions.

## 1. Introduction

The appearance of clinical symptoms leading to a loss of autonomy in neurodegenerative disorders takes about 15 years after the development of brain physiopathological lesions. This leaves a large time-window to initiate preventive treatments to slow down cognitive decline, or hopefully impede the onset of Alzheimer's disease (AD) (Bhatti et al., 2020). Still, the most impactful prevention strategies and the right populations to be targeted remain to be identified. Clinical trials have evaluated the impact of multiple interventions on older adults' cognition, including physical activity, cognitive stimulation, mediterranean diet, cardiovascular prevention, or nutritional supplementation (Brini et al., 2018; Kivipelto et al., 2018; Bott et al., 2019; Buckinx and Aubertin-Leheudre, 2021). Nutritional interventions have focused on multiple nutrients, although some supplements, such as omega-3 fatty acids, have received particular attention. Indeed, they show protective effects against age-related processes such as neuroinflammation (Joffre et al., 2020), oxidative stress (Mora et al., 2022), and blood-brain barrier dysfunction (Barnes et al., 2021). Omega-3 fatty acid supplementation has been associated with reduced memory (Yurko-Mauro et al., 2015) and cognitive (Marti del Moral and Fortique, 2019) impairment and a reduced risk of developing dementia (Zhang et al., 2015). More recently, various studies have evaluated the impact of multidomain preventive strategies, that combine different interventions, as it has been hypothesized that their effect might be optimal as they simultaneously target multiple risk factors associated with AD (Kivipelto et al., 2018). Several studies suggest that multi-domain interventions are indeed more effective than single-domain interventions on the cognition of older adults with mild cognitive impairment (MCI) (Salzman et al., 2022). Positive results from the FINGER trial have prompted the creation of a global initiative to evaluate and optimize the effect of multidomain lifestyle interventions (Word-Wide FINGERS) (Kivipelto et al., 2020). If promising, the results of interventional studies are however disparate (Solomon et al., 2021). In addition, the question of how interventions impact the brain remains unanswered.

Magnetic resonance imaging (MRI) of the brain can reveal localized, subtle and functional alterations. Anatomical MRI data has been predominantly used to evaluate interventions related with physical activity (Haeger et al., 2019), multidomain interventions (Stephen et al., 2019), as well as diets and/or effect of nutrition patterns on the brain (Bos et al., 2016; Rodrigues et al., 2020). Taken together, they indicate for instance that omega-3 fatty acid supplementation, nutritional patterns or red blood cell levels are associated with larger brain (Conklin et al., 2007; Pottala et al., 2014; Witte et al., 2014; Berti et al., 2015; Prinelli et al., 2019) and hippocampus (Samieri et al., 2012; Pottala et al., 2014; Witte et al., 2014) volumes and with increased white matter integrity (Tan et al., 2012; Virtanen et al., 2013; Witte et al., 2014). More recently, resting-state functional MRI (rs-fMRI) has been identified as a biomarker of interest. It characterizes brain regions functional similarity, and can describe neurodegenerescence across the continuum of AD (Hohenfeld et al., 2018), as well as brain alterations in the early asymptomatic phase of subjective cognitive decline (Viviano and Damoiseaux, 2020). Moreover, functional connectivity (FC) in middle-aged or older adults has been shown to be impacted by interventions. It is modified by physical activity (Chen et al., 2020), and can be altered by nutrition (Rodrigues et al., 2020). Omega-3 fatty acid nutritional patterns have for instance been associated with enhanced functional networks efficiency (Zwilling et al., 2019), and differences in FC have been associated with differences in omega-3 fatty acid red blood cell levels (Talukdar et al., 2019) and supplementation (Park et al., 2020). Cognitive training also modifies neural networks related to important cognitive functions. It induces opposing cognitive patterns to the ones typically associated with aging and neuro-degeneration. On the one hand, it increases the within-network connectivity of the Default Mode Network (DMN) and on the other, the anticorrelation between the DMN and the frontoparietal network (FPN) (van Balkom et al., 2020).

Despite these promising results, no study has yet addressed the impact of a multidomain intervention combining physical activity, cognitive training, nutritional counseling and an omega-3 fatty acid supplementation on the FC of an older population with cognitive complaints and thus at-risk to develop AD. Such intervention has been implemented in the Multidomain Alzheimer Preventive Trial (MAPT) (Vellas et al., 2014). It showed no effect on the cognitive decline of older adults (>70 years' old) at-risk for AD (Andrieu et al., 2017). We aim to decipher whether, despite this apparent lack of effect on cognition, the intervention may impact brain FC, which is a sensitive marker of brain integrity (Charroud et al., 2016; Conti et al., 2021).

We therefore aim:

- To investigate the impact of a multidomain preventive intervention on the FC of MAPT trial participants using whole brain rs-MRI data analysis;
- To study the effect of the intervention in subgroups defined by specific patient characteristics such as clinical dementia rating score (CDR; CDR = 0 vs. CRD = 0.5) or Fried's frailty criteria (Fried et al., 2004).

## 2. Materials and methods

### 2.1. General study design

All participants were recruited from the ancillary MAPT MRI study, which is fully described elsewhere (Vellas et al., 2014). Briefly, participants with either spontaneous memory complaint, limitation in one instrumental activity of daily living (IADL), slow gait speed ( ≤ 0.8 m/s), or a combination of these factors, were included in a multi-center randomized controlled trial designed to assess the efficacy of omega-3 fatty acid supplementation (Om3) and multidomain intervention (MI) alone, or in combination (Om3 + MI) against a placebo (Pl). The multidomain intervention combined cognitive training, physical activity and nutritional counseling (e.g., recommendation to increase fruit and vegetable consumption) (Hercberg et al., 2008), and was applied during 36 months. Omega-3 fatty acid or placebo supplementations were delivered daily during 36 months: participants consumed two capsules containing either 400 mg docosahexaenoic acid (DHA) and a maximum amount of 112.5 mg per capsule of eicosapentenoic acid (EPA) or a placebo. Three hundred and eighty participants underwent MRI imaging, demographical, clinical and cognitive evaluations at baseline that were repeated at 36 months (M36). Participants were excluded from the trial if they were diagnosed with (a) dementia, (b) a Mini Mental State Examination (MMSE) score (Folstein et al., 1975) lower than 24, (c) any difficulty in basic living activity or (d) if they were already taking an omega-3 fatty acids supplementation. The demographic characteristics included, amongst others, age, sex, and educational level. Intervention efficacy was primarily assessed in the parent study by using a cognitive composite score. Participant subgroups were defined by different risk profiles at baseline (Andrieu et al., 2017). The MAPT study protocol was approved by the Advisory Committee for the Protection of Persons participating in Biomedical Research of the Toulouse University Hospital, and was authorized by the French Health Authority. The protocol (NCT00672685) can be found on a public access clinical trial database (www.clinicaltrials.gov). This ancillary study was approved by the scientific committee of the MAPT study group.

### 2.2. Specific MRI based data selection and acquisition

**Figure 1:**
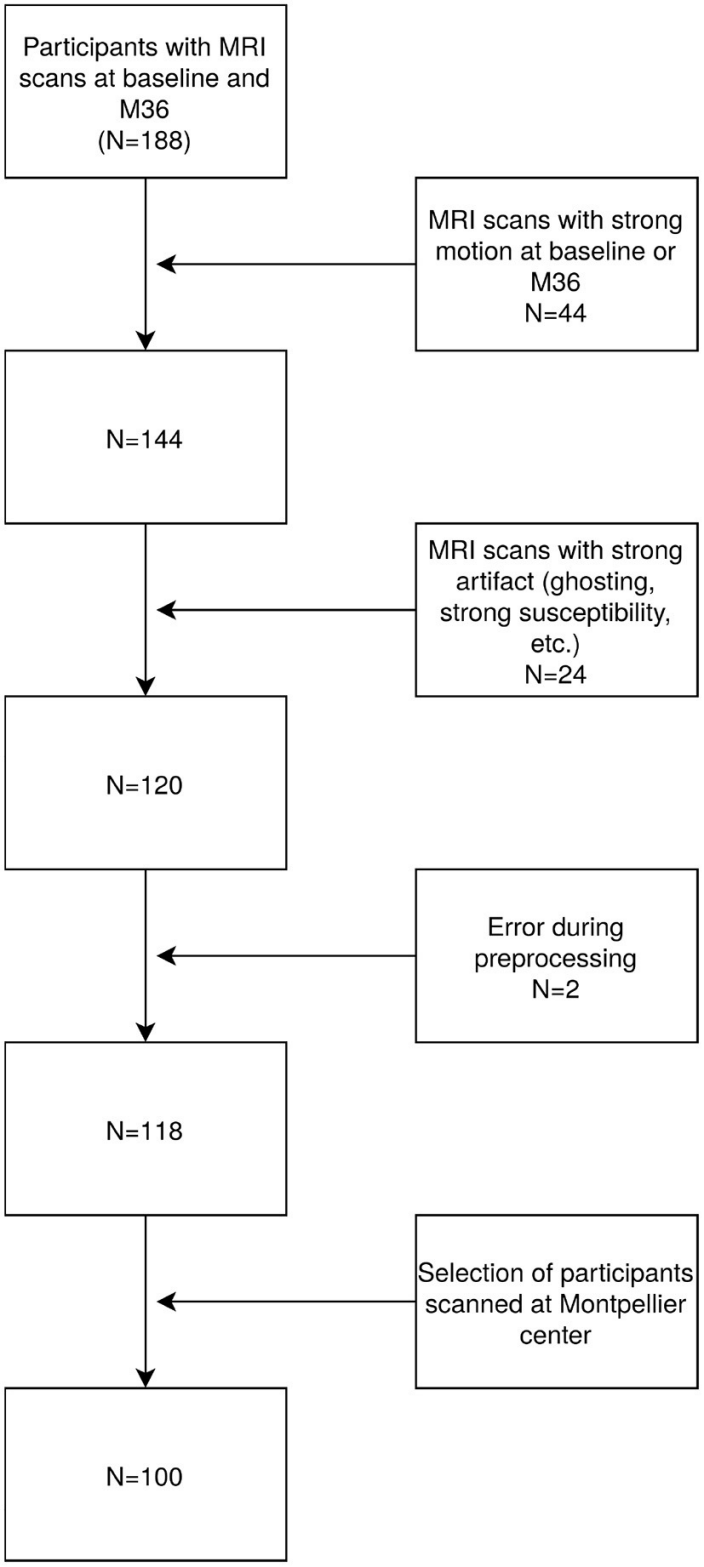
Chart of participants exclusion process.

The MAPT-MRI study included 188 participants with rs-fMR scans available at both baseline and follow-up (M36). On average, the baseline scan was performed at 114 days (3–569) after the beginning of the intervention. The follow-up scan was performed on average at 986 days (695–1388) after the first MRI scan. Only participants passing the rs-fMRI quality control were included for further analysis. Quality control included a combined analysis of automatic metrics and visual inspection (head coverage, intensity inhomogeneity, ghosting artifacts, etc.). Motion was evaluated using Framewise Displacement (FD) and DVARS metrics to characterize volumes with excessive motion, as suggested by Power et al. (2012). Their respective thresholds were 0.2 mm and 0.5%ΔBOLD signal change. Images with more than 15% of volumes exceeding both of these thresholds were discarded, as were images exceeding a threshold of 2 degrees of rotation or a translation of more than a voxel size. We excluded 44 participants with strong motion, 24 with other artifacts, two with failed preprocessing and finally we included only participants scanned in Montpellier to eliminate biases induced by artificial differences like scanner type and center effects. This resulted in a total of 100 participants being eligible for further evaluation (see the study flowchart, Figure 1).

All imaging was performed with an 1.5T Siemens AVANTO scanner. rs-fMRI parameters were: voxel size = 3 × 3 × 5 mm^3^, repetition time (TR) = 2,400 ms, echo time (TE) = 50 ms, flip angle = 90° and slice number = 28. The scanning lasted for 8.07 min. Anatomical 3DT1 MPRAGE parameters were: voxel size = 1 × 1 × 1 mm, TR = 2,100 s, TE = 1.4 ms, flip angle = 15°, inversion time (TI) = 1,100 ms, and slice number = 160. FLAIR sequence acquisition parameters were: voxel size = 0.9 × 0.9 × 5 mm, TR = 8s, TE = 109 ms, TI = 2.5 s, Turbo Factor = 21, and slice number = 27.

### 2.3. Resting-state functional MRI data preprocessing

Resting-state functional MRI data was preprocessed using the SPM12 toolbox (https://www.fil.ion.ucl.ac.uk/spm/). The preprocessing steps were as follows: the first 10 volumes of the functional images were discarded to reach a steady-state, then we performed subsequently slice time correction, motion correction with a six-parameters rigid-body spatial transformation, normalization to MNI space, and smoothing with a 6 mm kernel. Anatomical images were segmented into white matter (WM), gray matter (GM) and cerebrospinal fluid (CSF). In addition, using the Conn toolbox (v19c) (Whitfield-Gabrieli and Nieto-Castanon, 2012), functional scans were de-spiked, and physiological artifacts (WM and CSF mean global signals) and residual subject movement (six motion parameters and their derivatives) were removed with linear detrending and a band-pass filter of (0.008, 0.09) Hz was applied.

### 2.4. Computation of functional connectivity metrics

FC scores were computed between networks of the Bootstrap Analysis of Stable Clusters (BASC)–Cambridge atlas (Urchs et al., 2015), using the Conn toolbox. Each score is defined as the Fisher-transformed bivariate correlation coefficient between a pair of networks' mean time series. The BASC Cambridge atlas is a multiresolution atlas with multiple levels of whole-brain network parcellations ranging from seven to 444 networks. The coarser parcellation with seven networks (R7) comprises well-defined large-scale networks, such as the DMN (Badhwar et al., 2017). We selected for this analysis the parcellation with 36 networks (R36–630 connections) (Urchs et al., 2015). This parcellation has notably been used by Badhwar et al. (2017) to accurately pinpoint regions associated with AD in a review of rs-fMRI studies. For the purpose of clarity, we refer to the R36 networks as “subnetworks," as they are finer entities than the well-known networks defined in R7. Furthermore, in order to name and define these subnetworks, we report the larger brain networks from R7 with which they overlap. For instance, a subnetwork of R36 which overlaps with the R7 DMN network will be qualified as “a subnetwork of the DMN." Note that the overlap between a R36 subnetwork and R7 network is not necessarily total and that a subnetwork can overlap with different networks from R7. In that case, we report the R7 network with which the R36 subnetwork overlaps the most. We also report the main anatomical structures (e.g., middle frontal gyrus) that compose the subnetworks to name them.

### 2.5. Statistical methodology for functional connectivity analyses

All main statistical analyses were computed using non-parametric cluster statistics on the 630 subnetwork to subnetwork connections in the Conn toolbox. The Spatial Pairwise Clustering (SPC) method, as implemented in Conn, was used to identify related sets of connections sharing similar effects. Briefly, depending on the test, *T* or *F* statistics are computed for all connections, resulting in a matrix of statistical values. All subnetworks from this matrix are then sorted automatically using a hierarchical clustering procedure (Bar-Joseph et al., 2001) based on functional and anatomical similarity, and all connections are thresholded with an individual connection threshold. The thresholded connections are gathered into sets of non-overlapping clusters, that are characterized by their mass, that is, the sum of squared statistics over all their connections. The distribution of cluster mass values under the null hypothesis is estimated using permutations iterations on the data, and each cluster mass is compared to this distribution, resulting in a cluster uncorrected *p*-value representing the likelihood of having a randomly-selected cluster with a similar or larger mass under the null hypothesis. Family-Wise Error (FWE) correction is applied to all individual cluster *p*-values, to control for false positives. Conn default individual connection threshold for the SPC method (*p* < 0.01) was applied and clusters were kept for *p*-FWE < 0.05. As specified above, the SPC method sorts input regions, here the R36 subnetworks, using a hierarchical clustering procedure based on functional and anatomical similarity. This measure of similarity and thus the clustering procedure depends on participants' data. In order to keep the same and most stable clustering for all statistical tests, we always used the clustering derived from data with all included subjects at both timepoints.

In cases when the Conn toolbox analyses revealed significant between-group differences or pairwise group differences needed to be assessed, we performed *post-hoc* analysis outside the toolbox. Functional connectivity values were extracted at baseline and 36 months for all significant connections as determined by the SPC method. *Post-hoc* analyses assessed changes of each connection from baseline to 36 months using R (version 3.6.1) multcomp package (version 1.4-10).

### 2.6. Statistical group analyses on functional connectivity

Participants' characteristics were described using mean (standard deviation) [min–max] for quantitative variables, and percentages for qualitative variables. The effects of the MAPT study interventions were assessed on the whole population using a repeated measure ANOVA adjusted for age, sex and level of education (binarized as < University level or ≥ University level). Significant results using the unadjusted model were reported in the Supplementary material.

Additional subgroup analyses were also performed, and interaction effects between interventions and risk-factor based subgroups were added to the previous model. Two criteria defined in a previous research work on the MAPT cohort (Andrieu et al., 2017) were examined to define these subgroups: the CDR status (CDR = 0 vs. CRD = 0.5), and on the Fried's frailty criteria (none vs. at least one frailty criteria) (Fried et al., 2004).

It should be mentioned that the previous research work on the MAPT cohort defined other subgroups (Andrieu et al., 2017). These subgroups were based on: the MMSE score, red blood cell DHA and EPA concentrations, dementia risk (CAIDE score) (Kivipelto et al., 2006), APOE ϵ4 genotype, brain Aβ load [florbetapir PET scan (Fleisher, 2011)]. We could not test an effect of interventions on these subgroups due to limited sample sizes. Characteristics of these subgroups are described in Supplementary Table S1.

### 2.7. Structural brain characteristics: Cortical thickness and white matter hyperintensities load

It has been reported that WMH load and cortical thickness can have an impact on resting state FC for MCI participants (Wang et al., 2020; Vettore et al., 2021). Both WMH load and cortical thickness were thus computed and analyzed to ensure that the observed effects on FC were not driven by these factors. Cortical thickness was computed when the BASC-Cambridge atlas subnetworks displayed significantly modified FC for the above-defined tests. All subnetworks were first resliced with the SPM12 toolbox to the anatomical 2iso MNI brain template provided by FSL v6.0.0 (Jenkinson et al., 2012). The brain template was then registered to Freesurfer “fsaverage" subject space and the registration was used to transform the resliced subnetwork images (Freesurfer v6) (Fischl and Dale, 2000). Participants' cortical thickness was computed with the Freesurfer recon-all pipeline at baseline and 36 months and mapped to the fsaverage surface. Subnetworks' cortical thickness was then extracted and averaged over left and right hemispheres for each participant and at both timepoints. Difference of cortical thickness between both timepoints was eventually computed. Freesurfer participants' GM segmentation and subnetworks' mapping to fsaverage surface were visually inspected. The WMH load was evaluated using the White matter Hyperintensities Automated Segmentation Algorithm (WHASA) on available FLAIR and 3DT1 data. See Samaille et al. (2012) for a full description of the WHASA method. Volumes of WMH were evaluated at baseline and 36 months and the difference of volume between both timepoints was computed. All participants WMH volumes were expressed relative to the total intracranial volume (TIV) at baseline. Visual quality control excluded participants with poor quality segmentation. The participants' cortical thickness data and WMH volumes were computed by the CATI Platform.

All statistical analyses on cortical thickness and WMH load were adjusted for age, sex and level of education and performed using permutation statistics (*N* = 5,000 permutations) with the Palm software (v119) (Winkler et al., 2014). For cortical thickness analyses, false discovery rate correction (*p*-FDR < 0.05) was applied to take into account the multiple tests on all subnetworks.

## 3. Results

### 3.1. Baseline characteristics of the participants

All one hundred participants [37% male, mean (SD [min–max]) age 74.3 (±3.74, [70–84]) years] described spontaneous memory complaints but were not limited in instrumental activities of daily living and in addition about 11% showed reduced walking speed (below or equal to 0.8 m/s). At baseline, the mean cognitive composite score was 0.16 (±0.51, [–1.60; 1.27]) and the mean MMSE score was 28.01 (±1.38, [24; 30]), with 87% scoring below 30 at baseline. Furthermore, 38% showed at least one frailty criteria and 52% had a baseline CDR score of 0. A detailed description is presented in Table 1. Note that some participants show a change in CDR score over time, especially in the placebo and Om3 arms with a CDR-score evolution from 0.5 to 0. No significant difference is observed between included and excluded participants concerning age, sex and level of education, but participants included improved more on average during 36 months on the MMSE and cognitive composite scores than excluded participants. There is however no difference on CDR status between included and excluded participants (see Supplementary Table S2).

**Table 1:** Whole MAPT MRI subsample characteristics and comparison between the different intervention groups.

	**MRI subsample (*N* = 100)**	**Om3 + MI (*N* = 27)**	**Om3 (*N* = 24)**	**MI (*N* = 24)**	**Pl (*N* = 25)**	*p*-value^*^
Age, years (mean, (SD)/range)	74.26 (3.74)/ [70.00; 84.00]	74.59 (4.01)/ [70.00; 83.00]	73.92 (3.34)/ [70.00; 81.00]	74.79 (3.83)/ [70.00; 82.00]	73.72 (3.58)/ [70.00; 84.00]	0.756
Sex (F)	63 (63%)	16 (59%)	18 (75%)	14 (58%)	15 (60%)	0.580
Education level (≥University level)	49 (49%)	10 (37%)	15 (62%)	10 (42%)	14 (56%)	0.231
**Composite score (mean, (SD)/range)**						
At baseline	0.16 (0.51)/ [–1.60; 1.27]	0.16 (0.52)/ [–0.84; 1.23]	0.22 (0.34)/ [–0.62; 0.93]	0.03 (0.66)/ [–1.60; 1.27]	0.23 (0.45)/ [–0.54; 1.24]	0.522
Difference from baseline to 36 months	0.14 (0.49)/ [–1.61; 1.11]	0.09 (0.46)/ [–1.26; 0.94]	0.29 (0.39)/ [–0.53; 1.11]	0.08 (0.49)/ [–1.61; 0.88]	0.09 (0.56)/ [–1.58; 1.05]	0.388
**Mini Mental State Examination (mean, (SD)/range), /30**						
At baseline	28.01 (1.38)/ [24.00; 30.00]	28.04 (1.37)/ [25.00; 30.00]	28.29 (1.24)/ [24.00; 30.00]	28.00 (1.29)/ [25.00; 30.00]	27.72 (1.54)/ [24.00; 30.00]	0.491
Difference from baseline to 36 months	0.44 (1.73)/ [–5.00; 6.00]	0.30 (1.54)/ [–4.00; 2.00]	0.41 (1.85)/ [–3.00; 6.00]	0.38 (1.87)/ [–5.00; 4.00]	0.68 (1.64)/ [–3.00; 5.00]	0.904
**Slow gait speed (** ≤ **0.8 m/s)**^*a*^	11 (11%)	2 (7%)	4 (17%)	2 (8%)	3 (12%)	0.719
**Exploratory subgroups**						
**Clinical dementia rating at baseline**						0.260
0	52 (52%)	15 (56%)	16 (67%)	11 (46%)	10 (40%)	
0.5	48 (48%)	12 (44%)	8 (33%)	13 (54%)	15 (60%)	
**Clinical dementia rating evolution from baseline to 36 months**						0.002
0– 0	36 (36%)	6 (22%)	14 (58%)	6 (25%)	10 (40%)	
0.5–0.5	25 (25%)	9 (33%)	1 (4%)	9 (38%)	6 (24%)	
0–0.5	16 (16%)	9 (33%)	2 (8%)	5 (21%)	0 (0%)	
0.5–0	23 (23%)	3 (11%)	7 (29%)	4 (17%)	9 (36%)	
**Fried's frailty criteria**						0.487
No frailty criteria	57 (60%)	16 (62%)	16 (73%)	13 (54%)	12 (52%)	
At least one frailty criteria	38 (40%)	10 (38%)	6 (27%)	11 (46%)	11 (48%)	

* Comparison between intervention groups. Kruskall–Wallis and Anova or Chi-2 tests were used for quantitative and qualitative variables respectively.

a All participants presented spontaneous memory complaints but none of them were impeded in instrumental activities of daily living.

Percentages were calculated with the number of participants for whom data was available for each variable. Percentages were rounded to the nearest value. This rounding can result in a loss of accuracy and the sum of the percentages may be close but not equal to 100%.

Om3 + MI, omega-3 fatty acid supplementation and multidomain intervention; Om3, omega-3 fatty acid supplementation; MI, multidomain intervention; Pl, placebo.

### 3.2. Main effects of intervention and interactions with risk-factor based subgroups on FC

No difference of FC was found between the intervention groups over time. There was neither a main effect of time, indicating that the FC of participants combined across intervention groups did not differ between baseline and after 36 months of intervention, nor a main effect of groups, showing that FC did not differ between groups at pre and post intervention states. No effect was found for the unadjusted model either.

However, a significant interaction between baseline CDR status and intervention groups over time was found (Table 2, see Supplementary Table S3 for unadjusted model). These connections associated the frontoparietal (FPN) subnetworks with subnetworks of the sensorimotor (SMN), salience (SN) and visual (VIS) networks. Subnetworks from the FPN were mainly centered on the middle frontal gyrus. The structure and main network affiliation of all subnetworks that are significant for this interaction and for all results reported thereafter are further described in Supplementary Table S4.

**Table 2:** Significant connections for interaction over 36 months between intervention group and baseline CDR status.

Individual connection statistics*	**Subnetwork (main network) 1**	**Subnetwork (main network) 2**
*F* = 6.05 *p* < 0.001	L/R Cun/SOG (VIS)	L/R MFG/MSFG (FPN)
*F* = 5.64 *p* = 0.001	L/R SMG/Post CG (SN)	L/R MFG/SMG (FPN)
*F* = 5.38 *p* = 0.002	L/R Pre CG (SMN)	L/R MFG/SMG (FPN)
*F* = 5.06 *p* = 0.003	L/R Sup Pre/Post CG (SMN)	L/R MFG/MSFG (FPN)
*F* = 4.90 *p* = 0.003	L/R Sup Pre/Post CG (SMN)	L/R MFG/SMG (FPN)
*F* = 4.85 *p* = 0.004	L/R Pre/Post CG (SMN)	L/R MFG/SMG (FPN)
*F* = 4.84 *p* = 0.004	L/R SPL (SN)	L/R MFG/SMG (FPN)
*F* = 4.60 *p* = 0.005	L/R SMG/Post CG (SN)	L/R MFG/MSFG (FPN)

^*^Results presented are significant after SPC FWE-cluster correction. All significant connections are part of the same cluster.

The model was adjusted for age, sex and level of education.

VIS, Visual network; FPN, Frontoparietal network; SN, Salience network; SMN, Sensorimotor network; L/R Cun/SOG, left/right cuneus/superior occipital gyrus; L/R MFG/MSFG, left/right middle frontal gyrus/superior frontal gyrus medial segment; L/R SMG/Post CG, left/right supramarginal gyrus/post central gyrus; L/R MFG/SMG, left/right middle frontal gyrus/supramarginal gyrus; L/R Pre CG, left/right pre central gyrus; L/R Sup Pre/post CG, left/right superior pre/post central gyrus; L/R Pre/Post CG, left/right pre/post central gyrus; L/R SPL, left/right superior parietal lobule; SPC, spatial pairwise clustering; FWE, family-wise error.

**Figure 2:**
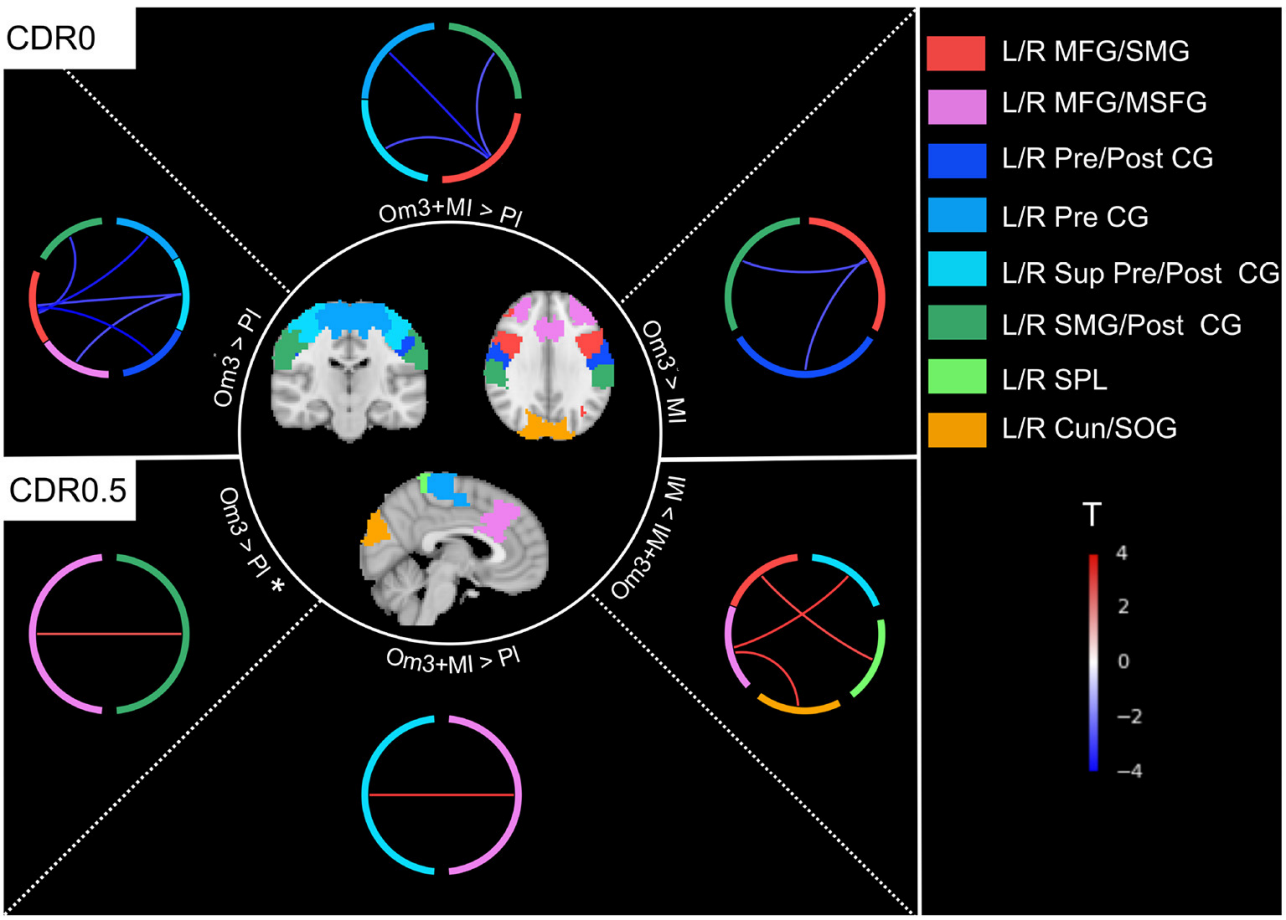
Connections showing a difference over the evolution of FC between interventions within each subgroup of baseline CDR0 and CDR0.5 (Table 3). Almost all connections were significant except for the L/R MFG/MSFG - L/R SMG/Post CG connection for the comparison CDR0.5 Om3 > Pl that was close to significance (*p* < 0.06)*. Om3 + MI, Omega-3 fatty acid supplementation and multidomain intervention; Om3, omega-3 fatty acid supplementation; MI, multidomain intervention; Pl, placebo; L/R Cun/SOG, left/right cuneus/superior occipital gyrus (visual network); L/R MFG/MSFG, left/right middle frontal gyrus/superior frontal gyrus medial segment (frontoparietal network); L/R SMG/Post CG, left/right supramarginal gyrus/post central gyrus (salience network); L/R MFG/SMG, left/right middle frontal gyrus/supramarginal gyrus (frontoparietal network); L/R Pre CG, left/right pre central gyrus (sensorimotor network); L/R Sup Pre/Post CG, left/right superior pre/post central gyrus (sensorimotor network); L/R Pre/Post CG, left/right pre/post central gyrus (sensorimotor network); L/R SPL, left/right superior parietal lobule (salience network); *T*, *t*-value for *post hoc* tests (Table 3), red connections represent a positive difference between mean connectivity of interventions group with omega-3 fatty acid supplementation vs. no omega-3 fatty acid supplementation, blue connections represent a negative difference.

*Post-hoc* analyses highlighted that FPN-SMN, FPN-SN and FPN-VIS connectivity was different between interventions with omega-3 fatty acid supplementation (Om3 and Om3 + MI) compared to interventions without omega-3 (MI and placebo), though this difference was dependant on participants baseline CDR status and displayed opposite directions between CDR0 and CDR0.5 subgroups of participants (Table 3, Figure 2). When examining directly the raw mean connectivity, it appeared that for participants with a CDR0 at baseline, the Om3 supplementation induced a stability or slight decrease of the FC compared to the placebo where FC was increased (Supplementary Table S3, Supplementary Figure S1). For the participants with CDR0.5 at baseline, FC also remained stable or increased after Om3 supplementation, but it systematically decreased more after placebo intake (Supplementary Table S3, Supplementary Figure S1). It is interesting to note that when we tested for an interaction between baseline CDR status and omega-3 fatty acid supplementation (Om3 and Om3 + MI) vs. no omega-3 fatty acid supplementation (MI and placebo), the same FPN-SMN, FPN-SN and FPN-VIS connections were significant, though new significant connections between the FPN-SMN, FPN-VIS, FPN-SN, DMN-VIS, and DMN-SMN were revealed (Supplementary Table S5). This shows that the observed effect between intervention groups and baseline CDR status is driven by omega-3 fatty acid supplementation. In contrast, no interaction effects were found between intervention groups and Fried's frailty criteria.

**Table 3:** ***Post hoc*** tests on significant connections between subnetworks (main networks) for interaction intervention group × baseline CDR status × time.

	**CDR0 at baseline (*****t*****;** ***p*** value^*^) **Om3** + **MI (N** = **15)** ≠ **Om3 (N** = **16)** ≠ **MI (N** = **11)** ≠ **Pl (N** = **10)**						**CDR0.5 at baseline (*****t*****;** ***p*** value^*^) **Om3** + **MI (N** = **12)** ≠ **Om3 (N** = **8)** ≠ **MI (N** = **13)** ≠ **Pl (N** = **15)**					
	**Om3 + MI > Om3**	**Om3 + MI > MI**	**Om3 > MI**	**Om3 + MI > Pl**	**Om3 > Pl**	**IM > Pl**	**Om3 + MI > Om3**	**Om3 + MI > MI**	**Om3 > MI**	**Om3 + MI > Pl**	**Om3 > Pl**	**IM > Pl**
**Connections from subnetwork L/R MFG/SMG (FPN) to subnetworks**												
L/R SMG/Post CG (SN)	*T*= 0.40 *p*= 0.978	*T*= –2.38 *p*= 0.094	***T* = ** **–2.80** ***p* = ** **0.036**	***T* = ** **–2.67** ***p* = ** **0.049**	***T* = ** **–3.10** ***p* = ** **0.017**	*T*= –0.35 *p*= 0.984	*T*= –0.14 *p*= 0.999	*T*= 1.18 *p*= 0.638	*T*= 1.36 *p*= 0.527	*T*= 1.42 *p*= 0.490	*T*= 1.36 *p*= 0.527	*T*= 0.22 *p*= 0.996
L/R SPL (SN)	*T*= 0.49 *p*= 0.961	*T*= –1.44 *p*= 0.478	*T*= –1.93 *p*= 0.231	*T*= –2.03 *p*= 0.190	*T*= –2.53 *p*= 0.069	*T*= –0.61 *p*= 0.928	*T*= 1.11 *p*= 0.685	***T* = ** **2.83** ***p* = ** **0.034**	*T*= 1.22 *p*= 0.617	*T*= 2.20 *p*= 0.139	*T*= 0.71 *p*= 0.891	*T*= –0.70 *p*= 0.894
L/R Pre CG (SMN)	*T*= –0.04 *p*= 1.000	*T*= –2.01 *p*= 0.199	*T*= –2.01 *p*= 0.197	***T* = ** **–3.52** ***p* = ** **0.005**	***T* = ** **3.58** ***p* = ** **0.004**	*T*= –1.50 *p*= 0.444	*T*= 1.03 *p*= 0.734	*T*= 1.83 *p*= 0.273	*T*= 0.48 *p*= 0.963	*T*= 1.91 *p*= 0.237	*T*= 0.55 *p*= 0.945	*T*= 0.05 *p*= 1.000
L/R Sup Pre/Post CG (SMN)	*T*= –0.05 *p*= 1.000	*T*= –2.28 *p*= 0.118	*T*= –2.28 *p*= 0.119	***T* = ** **–2.88** ***p* = ** **0.029**	***T* = ** **–2.92** ***p* = ** **0.027**	*T*= –0.66 *p*= 0.912	*T*= 1.70 *p*= 0.334	*T*= 1.59 *p*= 0.392	*T*= –0.38 *p*= 0.981	*T*= 2.22 *p*= 0.133	*T*= 0.10 *p*= 1.000	*T*= 0.60 *p*= 0.929
L/R Pre/Post CG (SMN)	*T*= 1.39 *p*= 0.509	*T*= –1.35 *p*= 0.533	***T* = ** **–2.67** ***p* = ** **0.049**	*T*= –2.5 *p*= 0.074	***T* = ** **–3.82** ***p* = ** **0.002**	*T*= –1.13 *p*= 0.672	*T*= 0.82 *p*= 0.842	*T*= 2.38 *p*= 0.097	*T*= 1.13 *p*= 0.674	*T*= 1.48 *p*= 0.453	*T*= 0.40 *p*= 0.978	*T*= –0.96 *p*= 0.773
**Connections from subnetwork L/R MFG/MSFG (FPN) to subnetworks**												
L/R Cun/SOG (VIS)	*T*= 0.32 *p*= 0.988	*T*= –1.87 *p*= 0.256	*T*= –2.20 *p*= 0.138	*T*= –1.91 *p*= 0.236	*T*= –2.21 *p*= 0.124	*T*= –0.11 *p*= 1.00	*T*= 0.54 *p*= 0.947	***T* = ** **2.97** ***p* = ** **0.024**	*T*= 1.89 *p*= 0.247	*T*= 2.06 *p*= 0.182	*T*= 1.18 *p*= 0.638	*T*= –0.99 *p*= 0.753
L/R Sup Pre/Post CG (SMN)	*T*= 0.75 *p*= 0.876	*T*= –1.02 *p*= 0.739	*T*= –1.73 *p*= 0.318	*T*= –2.05 *p*= 0.185	***T* = ** **–2.78** ***p* = ** **0.038**	*T*= –1.01 *p*= 0.742	*T*= 1.75 *p*= 0.310	***T* = ** **3.05** ***p* = ** **0.020**	*T*= 0.76 *p*= 0.869	***T* = ** **3.10** ***p* = ** **0.017**	*T*= 0.81 *p*= 0.850	*T*= –0.01 *p*= 1.000
L/R SMG/Post CG (SN)	*T*= 0.67 *p*= 0.908	*T*= –0.78 *p*= 0.865	*T*= –1.41 *p*= 0.497	*T*= –1.45 *p*= 0.476	*T*= –2.09 *p*= 0.171	*T*= –0.66 *p*= 0.909	*T*= –1.23 *p*= 0.610	*T*= 0.99 *p*= 0.756	*T*= 2.01 0.198	*T*= 1.57 *p*= 0.402	***T* = ** **2.63** ***p* = ** **0.055**	*T*= 0.57 *p*= 0.938

Significant results are outlined in bold (near significant are in bold and italics).

* The model was adjusted for age, sex and level of education.

Om3 + MI, omega-3 fatty acid supplementation and Multidomain intervention; Om3, omega-3 fatty acid supplementation; MI, multidomain intervention; Pl, placebo; VIS, Visual network; FPN, Frontoparietal network; SN, Salience network; SMN, Sensorimotor network; L/R Cun/SOG, left/right cuneus/superior occipital gyrus; L/R MFG/MSFG, left/right middle frontal gyrus/superior frontal gyrus medial segment; L/R SMG/Post CG, left/right supramarginal gyrus/post central gyrus; L/R MFG/SMG, left/right middle frontal gyrus/supramarginal gyrus; L/R Pre CG, left/right pre central gyrus; L/R Sup Pre/Post CG, left/right superior pre/post central gyrus; L/R Pre/Post CG, left/right pre/post central gyrus; L/R SPL, lef/right superior parietal lobule.

### 3.3. CDR status and omega-3 fatty acid supplementation

#### 3.3.1. The effect of CDR status and omega-3 fatty acid supplementation on cortical thickness and WMH load: Implications for FC

Brain structural alterations may drive the differences of FC observed between participants with different CDR status and with or without an omega-3 fatty acid supplementation. To explain the observed interaction effect between omega-3 fatty acid supplementation and CDR status, we first tested whether the FC changes of the FPN with the SMN, SN and VIS networks (Table 2) could be explained by differences of cortical thickness between the groups. For each subnetwork, the evolution of cortical thickness between baseline and 36 months was compared between CDR0 participants supplemented with Om3 (Om3 and Om3 + MI: *N* = 29) and without (MI and placebo: *N* = 20) and between CDR0.5 participants supplemented with Om3 (Om3 and Om3 + MI: *N* = 20) and without (*N* = 28) after exclusion of participants that did not reach quality control for cortical thickness analysis. The same evaluation was performed for the evolution of WMH load between CDR0 participants supplemented with Om3 (Om3 and Om3 + MI: *N* = 26) and without (MI and placebo: *N* = 17) and between CDR0.5 participants supplemented with Om3 (Om3 and Om3 + MI: *N* = 17) and without (*N* = 23) after excluding participants with erroneous WMH segmentation. No difference was detected, suggesting that these structural characteristics do not drive the FC changes observed for the CDR0 or CDR0.5 participants supplemented with Om3.

#### 3.3.2. Baseline differences in FC between omega-3 fatty acid and placebo supplementations according to participants' CDR status

We observed an effect of intervention linked to baseline CDR status on FC evolution. To determine if this effect could be attributed to baseline FC differences, we assessed the interaction between intervention groups and baseline CDR status on baseline FC. No significant difference was found, implying that FC is not different at baseline according to CDR status and group assignment. There was neither a significant difference when we pooled participants into larger groups of subjects supplemented or not with omega-3 fatty acids (Om3 & Om3 + MI vs. MI & placebo) and tested for an interaction with baseline CDR status on baseline FC.

#### 3.3.3. The role of the progression of the CDR on FC

Two profiles may exist: stable cognitive participants with a CDR0 or CDR0.5 at both time-points and participants that show cognitive changes, marked by a CDR0 at baseline and CDR0.5 at follow-up or vice-versa (Table 1). No significant difference on the evolution of FC was found after testing the effect of Om3 supplementation on subgroups of participants with stable CDR0 or CDR0.5 status. There was however a trend for stable CDR0 participants (*p* = 0.067; Supplementary Table S6) for the same FPN-SMN and FPN-SN connections that were significant when testing the effect of Om3 supplementation in CDR0 participants (Table 3). This suggests that this subset of stable participants may be equally responsive to Om3 intake. A trend for participants with stable CDR0.5 (*p* = 0.078; Supplementary Table S7) was also detected, but on VIS-SN connections that differed from the ones previously observed (Table 2). We thus cannot conclude if we observe a true effect of Om3 intake on stable CDR0.5 participants.

## 4. Discussion

This study aimed to evaluate the impact of preventive uni or multidomain intervention strategies during a 3-years period on older people with cognitive complaints by analyzing longitudinal FC changes. While reasonably large imaging data sample that included quality control procedures was assessed, no FC changes could be revealed for any intervention (MI including physical activity, cognitive training, and nutritional counseling alone, vs. omega-3 fatty acid supplementation alone, and MI combined with omega-3 fatty acid supplementation) . When targeting cognitive status at baseline, interestingly, an effect of the omega-3 fatty acid supplementation on longitudinal FC emerged, suggesting an effect of this supplementation on selected populations.

Participants with a normal CDR at baseline had stable FPN-VIS, FPN-SMN, and FPN-SN FC through time when receiving omega-3 fatty acid supplementation, while their FC would naturally increase without this supplementation (i.e., in the placebo group). This observation was also partially confirmed for the subset of participants with a stable good cognitive profile over time (CDR0 at baseline and follow-up), endorsing the fact that this process is also associated with a good prognosis. For participants with slight cognitive impairment (CDR0.5) the FPN-SMN, FPN-VIS and FPN-SN FC either remained stable or slightly increased with omega-3 fatty acid supplementation, while it decreased with placebo intake.

For participants with normal CDR, the lack of omega-3 fatty acid supplementation induces an increased between-network connectivity that is similar to what is observed in the aging process (Betzel et al., 2014; Geerligs et al., 2015). This could be imputed to either a loss of functional specificity from the different resting-state networks, or to a compensatory process in which networks act conjointly to maintain stable cognitive functions within time (Jockwitz and Caspers, 2021). In this light, omega-3 fatty acid supplementation could stabilize FC between networks. For participants with impaired cognition (CDR0.5), FC evolves differently. Viviano and Damoiseaux (2020) note in their review that various and inconsistent results about the FC of populations with subjective memory complaints have been reported, and propose a nonlinear model of FC evolution to explain this variability, though specifically for DMN and medial temporal regions. FC would first increase, due to noisy signals or compensatory mechanisms, to later decrease as neurodegeneration spreads. It could be hypothesized that CDR0.5 participants are either at the beginning or in the middle of this latter phase of decline. They would, when ingesting the placebo and depending on the connection, either have stable or decreasing between-network FC whereas omega-3 fatty acid supplementation would result in an opposite process by respectively increasing or preserving inter network connectivity. This interpretation should be taken with caution, as the model proposed by Viviano et al. is specific to the DMN and medial temporal regions and may not apply to other regions or networks.

The mechanisms linked to omega-3 fatty acid supplementation are not fully understood and do not seem in our study to be modulated by the cortical thickness nor the WMH load. An absence of change in cortical thickness was not expected as omega-3 fatty acid supplementation or fish-oil supplements have been shown to reduce atrophy in the temporal and parietal cortex, and more particularly in the hippocampus, for healthy older adults (Conklin et al., 2007; Raji et al., 2014; Witte et al., 2014; Daiello et al., 2015) and MCI and AD participants (Daiello et al., 2015). In addition, natural red blood cell DHA and EPA concentrations equally reshape GM in healthy older people (Walhovd, 2014). Concerning FC, a direct association was found between these omega-3 blood cell levels and FC of regions including the prefrontal cortex, hippocampus, precuneus and amygdala (Talukdar et al., 2019). When assessed indirectly through nutrient patterns, omega-3 fatty acid supplementation impacted the FC of the visual network and influenced the relation between the FC of the FPN and intelligence (Zwilling et al., 2019). The modifications we observe in FC of regions centered around the middle frontal gyrus and subnetworks of the FPN are congruent with the hypothesis that frontal regions are affected by cognitive impairment and neurodegenerative disease, like AD (Bayram et al., 2018).

We did not find any effect of the MI, alone or combined with omega-3 fatty acid supplementation for the overall population or risk-based subgroups. Interestingly, when looking at the main MAPT study as well as other brain imaging studies on MI, mixed results are reported. The ancillary FDG PET MAPT Trial showed that MI had no impact on global brain metabolism at 12 months (Delrieu et al., 2020). However, an effect on brain morphometry was detected through a specific deformation-based method, whereas classic segmentation based analyses did not yield any result (Sivera et al., 2020). This underscores the importance of methodological choices for the analysis of brain imaging data. Other trials such as the FINGER and PREDIVA studies failed to detect an effect of MI on either gray matter structure (Stephen et al., 2019) or WMH volumes (van Dalen et al., 2017; Stephen et al., 2019). They however showed that these interventions were more beneficial for participants with higher baseline cortical thickness in regions affected by AD (Stephen et al., 2019), or with higher baseline WMH volumes (van Dalen et al., 2017). This is concordant with our findings which suggest that the effect of interventions on the brain may be dependent on participants' baseline profiles.

The differences between studies examining the effect of preventive interventions and our findings, whether it concerns the effect of omega-3 fatty acid supplementation associated with participants' CDR status or the lack of benefit from MI, can be explained by multiple factors. First, inclusion criteria differed between studies, leading to populations with different cognitive profiles (cognitively healthy, at-risk to develop AD, frail, early AD, etc). Furthermore, beyond cognitive status, there is variability in the response of individuals to interventions, and many factors can modulate the effect of an intervention. Previous analyses suggest for instance that the impact of exercise on older people with mild AD depends on their APOE genotype (Jensen et al., 2019) or that the effect of omega-3 fatty acid supplementation on cognitively impaired older adults is influenced by participants' baseline plasma homocysteine levels (Jernerén et al., 2019). Second, the methodological choices to evaluate MRI data metrics and in particular FC varied between studies. It is instructive to note that in their review on cognitive training, van Balkom et al. (2020) identify the use of seed-based approaches as one of the major limitations in FC studies, as the results are highly dependent on the selected source seed. Similarly, the choice of brain atlas used to parcelate the brain might bias findings. Here we used one parcellation with 36 regions (630 connections) from a multiresolution atlas that was previously used in a meta-analysis to identify precise regions affected by AD (Badhwar et al., 2017). Other methodological choices concerning the integration of confounding risk factors such as APOEϵ4 or amyloid brain deposits can also explain the variability observed between studies (Rakesh et al., 2017; Bhatti et al., 2020; Yao et al., 2020). Eventually, differences between studies can be attributed to the intervention design that may vary in length, modality, frequency, and intensity. Also, it should be mentioned that while we mostly compare our findings to the results of interventional studies, some of the studies concerning omega-3 fatty acids are observational. Within our study, training intensity for MI differed over three-years. Participants underwent twelve sessions during the first two months, but this frequency was then reduced to one session per month. It could be argued that by the time participants were scanned at follow-up, any impact of the initial high intensity intervention had been wiped out, and that effects could have been observed at an earlier stage of the intervention. We did detect, in contrast, an effect of omega-3 fatty acid supplementation, and it is interesting to note that this specific intervention intensity was maintained during the three-year period.

This study present strengths but also limits. The study strength includes a substantial amount of MRI data, a quality control of imaging data, multimodal data, a longitudinal design and the complementary nature of MI. Some limitations should however be considered. Missing data (17%) might have limited the interpretation of WMH load effects on FC. Also, the small number of subjects in the subgroup analyses (CDR, Fried's frailty criteria), the even smaller number of subjects for some subgroups (APOE ϵ4 carriers, participants with low levels of omega-3, with high risk dementia, with low MMSE score) and missing information (amyloid status) unfortunately limited further detailed analysis.

Our study should be interpreted in the light of what is known about the amyloid status. Longitudinal analyses pointed out that the evolution of connectivity for cognitively intact older adults differed according to their amyloid status (Lin et al., 2020) and amyloid status appeared to modify FC over a wide range of studies including participants in the spectrum of AD (Hasani et al., 2021). Participants' amyloid load may thus be an important factor mediating the effect of interventions on FC. In our case, we reasoned that intervention groups presented similar cognitive profiles at baseline and were homogeneous on factors such as APOE ϵ4 status that are associated with increased amyloid burden (Sánchez-Juan and Seshadri, 2017; Sperling et al., 2020; Janssen et al., 2021). We hypothesize that it should limit the differences of amyloid load between the groups. Given the low number of APOE ϵ4 subjects in the sample it is also possible that few participants had a high amyloid burden. Nevertheless, the information about amyloid status should be taken into account in future studies.

In addition to replicating the results on CDR status, future studies on preventive interventions should aim to explore the effects of interventions on MRI biomarkers in potential subgroups of interest and to scan large samples of participants to this effect.

## 5. Conclusion

Older people with cognitive complaints did not show any change in FC after 36 months of multidomain or unidomain interventions. However an effect of omega-3 fatty acid supplementation on the FC of the frontoparietal, sensorimotor, visual and salience networks was detected if participants cognitive decline was considered. This effect was located on the frontoparietal network, known to be involved in neurodegenerative and aging processes, and showed opposite patterns of connectivity for participants with CDR0 or CDR0.5 at baseline. This effect was independent of cortical thickness and WMH load. Overall, our findings suggest that preventive strategies should consider participants' cognitive status and the heterogeneity of target populations when designing future studies on preventive interventions.

## Data availability statement

The original contributions presented in the study are included in the article/Supplementary material, further inquiries can be directed to the corresponding author.

## Ethics statement

The studies involving human participants were reviewed and approved by the Advisory Committee for the Protection of Persons participating in Biomedical Research of the Toulouse University Hospital and authorized by the French Health Authority. The patients/participants provided their written informed consent to participate in this study.

## MAPT/DSA group

###  MAPT study group

Principal investigator: Bruno Vellas (Toulouse); Coordination: Sophie Guyonnet; Project leader: Isabelle Carrié; CRA: Lauréne Brigitte; Investigators: Catherine Faisant, Françoise Lala, Julien Delrieu, Hélène Villars; Psychologists: Emeline Combrouze, Carole Badufle, Audrey Zueras; Methodology, statistical analysis and data management: Sandrine Andrieu, Christelle Cantet, Christophe Morin; Multidomain group: Gabor Abellan Van Kan, Charlotte Dupuy, Yves Rolland (physical and nutritional components), Céline Caillaud, Pierre-Jean Ousset (cognitive component), Françoise Lala (preventive consultation). The cognitive component was designed in collaboration with Sherry Willis from the University of Seattle, and Sylvie Belleville, Brigitte Gilbert, and Francine Fontaine from the University of Montreal.

Co-investigators in associated centers: Jean-François Dartigues, Isabelle Marcet, Fleur Delva, Alexandra Foubert, Sandrine Cerda (Bordeaux); Marie-Noëlle-Cuffi, Corinne Costes (Castres); Olivier Rouaud, Patrick Manckoundia, Valérie Quipourt, Sophie Marilier, Evelyne Franon (Dijon); Lawrence Bories, Marie-Laure Pader, Marie-France Basset, Bruno Lapoujade, Valérie Faure, Michael Li Yung Tong, Christine Malick-Loiseau, Evelyne Cazaban-Campistron (Foix); Françoise Desclaux, Colette Blatge (Lavaur); Thierry Dantoine, Cécile Laubarie-Mouret, Isabelle Saulnier, Jean-Pierre Clément, Marie-Agnès Picat, Laurence Bernard-Bourzeix, Stéphanie Willebois, Iléana Désormais, Noëlle Cardinaud (Limoges); Marc Bonnefoy, Pierre Livet, Pascale Rebaudet, Claire Gédéon, Catherine Burdet, Flavien Terracol (Lyon), Alain Pesce, Stéphanie Roth, Sylvie Chaillou, Sandrine Louchart (Monaco); Kristel Sudres, Nicolas Lebrun, Nadège Barro-Belaygues (Montauban); Jacques Touchon, Karim Bennys, Audrey Gabelle, Aurélia Romano, Lynda Touati, Cécilia Marelli, Cécile Pays (Montpellier); Philippe Robert, Franck Le Duff, Claire Gervais, Sébastien Gonfrier (Nice); Yannick Gasnier and Serge Bordes, Danièle Begorre, Christian Carpuat, Khaled Khales, Jean-François Lefebvre, Samira Misbah El Idrissi, Pierre Skolil, Jean-Pierre Salles (Tarbes).

MRI group: Carole Dufouil (Bordeaux), Stéphane Lehéricy, Marie Chupin, Jean-François Mangin, Ali Bouhayia (Paris); Michèle Allard (Bordeaux); Frédéric Ricolfi (Dijon); Dominique Dubois (Foix); Marie Paule Bonceour Martel (Limoges); François Cotton (Lyon); Alain Bonafé (Montpellier); Stéphane Chanalet (Nice); Françoise Hugon (Tarbes); Fabrice Bonneville, Christophe Cognard, François Chollet (Toulouse).

PET scans group: Pierre Payoux, Thierry Voisin, Julien Delrieu, Sophie Peiffer, Anne Hitzel, (Toulouse); Michèle Allard (Bordeaux); Michel Zanca (Montpellier); Jacques Monteil (Limoges); Jacques Darcourt (Nice). Medico-economics group: Laurent Molinier, Hélène Derumeaux, Nadège Costa (Toulouse). Biological sample collection: Bertrand Perret, Claire Vinel, Sylvie Caspar-Bauguil (Toulouse). Safety management: Pascale Olivier-Abbal.

DSA group: Sandrine Andrieu, Christelle Cantet, Nicola Coley.

## Author contributions

LP, JD, L-AG, EG, CF, and EL contributed to data analyses and manuscript writing. J-FM, CM, GB, LV, SG, BV, and AG contributed to manuscript writing. All authors contributed to the article and approved the submitted version.

## References

[B1] Andrieu S. Guyonnet S. Coley N. Cantet C. Bonnefoy M. Bordes S.. (2017). Effect of long-term omega 3 polyunsaturated fatty acid supplementation with or without multidomain intervention on cognitive function in elderly adults with memory complaints (MAPT): a randomised, placebo-controlled trial. Lancet Neurol. 16, 377–389. 10.1016/S1474-4422(17)30040-6 28359749

[B2] Badhwar A. Tam A. Dansereau C. Orban P. Hoffstaedter F. Bellec P.. (2017). Resting-state network dysfunction in Alzheimer's disease: a systematic review and meta-analysis. Alzheimers Dement. 8, 73–85. 10.1016/j.dadm.2017.03.007 28560308 PMC5436069

[B3] Bar-Joseph Z. Gifford D. K. Jaakkola T. S. (2001). Fast optimal leaf ordering for hierarchical clustering. Bioinformatics 17, S22–S29. 10.1093/bioinformatics/17.suppl_1.S22 11472989

[B4] Barnes S. Chowdhury S. Gatto N. M. Fraser G. E. Lee G. J. (2021). Omega-3 fatty acids are associated with blood?brain barrier integrity in a healthy aging population. Brain Behav. 11, e2273. 10.1002/brb3.2273 34327870 PMC8413753

[B5] Bayram E. Caldwell J. Z. Banks S. J. (2018). Current understanding of magnetic resonance imaging biomarkers and memory in Alzheimer's disease. Alzheimers Dement. 4, 395–413. 10.1016/j.trci.2018.04.007 30229130 PMC6140335

[B6] Berti V. Murray J. Davies M. Spector N. Tsui W. H. Li Y.. (2015). Nutrient patterns and brain biomarkers of Alzheimer's disease in cognitively normal individuals. J. Nutr. Health Aging 19, 413–423. 10.1007/s12603-014-0534-0 25809805 PMC4375781

[B7] Betzel R. F. Byrge L. He Y. Goñi J. Zuo X.-N. Sporns O. (2014). Changes in structural and functional connectivity among resting-state networks across the human lifespan. Neuroimage 102, 345–357. 10.1016/j.neuroimage.2014.07.067 25109530

[B8] Bhatti G. K. Reddy A. P. Reddy P. H. Bhatti J. S. (2020). Lifestyle Modifications and nutritional interventions in aging-associated cognitive decline and Alzheimer's disease. Front. Aging Neurosci. 11, 369. 10.3389/fnagi.2019.00369 31998117 PMC6966236

[B9] Bos D. J. van Montfort S. J. Oranje B. Durston S. Smeets P. A. (2016). Effects of omega-3 polyunsaturated fatty acids on human brain morphology and function: what is the evidence? Eur. Neuropsychopharmacol. 26, 546–561. 10.1016/j.euroneuro.2015.12.031 26742901

[B10] Bott N. T. Hall A. Madero E. N. Glenn J. M. Fuseya N. Gills J. L.. (2019). Face-to-face and digital multidomain lifestyle interventions to enhance cognitive reserve and reduce risk of Alzheimer's disease and related dementias: a review of completed and prospective Studies. Nutrients 11, 2258. 10.3390/nu11092258 31546966 PMC6770494

[B11] Brini S. Sohrabi H. R. Peiffer J. J. Karrasch M. Hämäläinen H. Martins R. N.. (2018). Physical activity in preventing Alzheimer's disease and cognitive decline: a narrative review. Sports Med. 48, 29–44. 10.1007/s40279-017-0787-y 28940148

[B12] Buckinx F. Aubertin-Leheudre M. (2021). Nutrition to prevent or treat cognitive impairment in older adults: a GRADE recommendation. J. Prev. Alzheimers Dis. 8, 7. 10.14283/jpad.2020.40 33336232

[B13] Charroud C. Le Bars E. Deverdun J. Steffener J. Molino F. Abdennour M.. (2016). Working memory performance is related to intrinsic resting state functional connectivity changes in community-dwelling elderly cohort. Neurobiol. Learn. Mem. 132, 57–66. 10.1016/j.nlm.2016.05.008 27234057

[B14] Chen F.-T. Hopman R. J. Huang C.-J. Chu C.-H. Hillman C. H. Hung T.-M.. (2020). The effect of exercise training on brain structure and function in older adults: a systematic review based on evidence from randomized control trials. J Clin. Med. 9, 914. 10.3390/jcm9040914 32230708 PMC7230405

[B15] Conklin S. M. Gianaros P. J. Brown S. M. Yao J. K. Hariri A. R. Manuck S. B.. (2007). Long-chain omega-3 fatty acid intake is associated positively with corticolimbic gray matter volume in healthy adults. Neurosci. Lett. 421, 209–212. 10.1016/j.neulet.2007.04.086 17574755

[B16] Conti L. Riccitelli G. C. Preziosa P. Vizzino C. Marchesi O. Rocca M. A.. (2021). Effect of cognitive reserve on structural and functional MRI measures in healthy subjects: a multiparametric assessment. J. Neurol. 268, 1780–1791. 10.1007/s00415-020-10331-6 33387014

[B17] Daiello L. A. Gongvatana A. Dunsiger S. Cohen R. A. Ott B. R. (2015). Association of fish oil supplement use with preservation of brain volume and cognitive function. Alzheimers Dement. 11, 226–235. 10.1016/j.jalz.2014.02.005 24954371 PMC4829435

[B18] Delrieu J. Voisin T. Saint-Aubert L. Carrie I. Cantet C. Vellas B.. (2020). The impact of a multi-domain intervention on cerebral glucose metabolism: analysis from the randomized ancillary FDG PET MAPT trial. Alzheimers Res. Ther. 12, 134. 10.1186/s13195-020-00683-6 33076983 PMC7574215

[B19] Fischl B. Dale A. M. (2000). Measuring the thickness of the human cerebral cortex from magnetic resonance images. Proc. Nat. Acad. Sci. 97, 11050–11055. 10.1073/pnas.200033797 10984517 PMC27146

[B20] Fleisher A. S. (2011). Using positron emission tomography and florbetapir F 18 to image cortical amyloid in patients with mild cognitive impairment or dementia due to Alzheimer disease. Arch. Neurol. 68, 1404. 10.1001/archneurol.2011.150 21747008

[B21] Folstein M. F. Folstein S. E. McHugh P. R. (1975). “Mini-mental state”. J. Psychiatr. Res. 12, 189–198. 10.1016/0022-3956(75)90026-6 1202204

[B22] Fried L. P. Ferrucci L. Darer J. Williamson J. D. Anderson G. (2004). Untangling the concepts of disability, frailty, and comorbidity: implications for improved targeting and care. J. Gerontol. A Biol. Sci. Med. Sci. 59, M255–M263. 10.1093/gerona/59.3.M255 15031310

[B23] Geerligs L. Renken R. J. Saliasi E. Maurits N. M. Lorist M. M. (2015). A brain-wide study of age-related changes in functional connectivity. Cereb. Cortex 25, 1987–1999. 10.1093/cercor/bhu012 24532319

[B24] Haeger A. Costa A. S. Schulz J. B. Reetz K. (2019). Cerebral changes improved by physical activity during cognitive decline: a systematic review on MRI studies. Neuroimage Clin. 23, 101933. 10.1016/j.nicl.2019.101933 31491837 PMC6699421

[B25] Hasani S. A. Mayeli M. Salehi M. A. Barzegar Parizi R. (2021). A systematic review of the association between amyloid-β and τ pathology with functional connectivity alterations in the Alzheimer dementia spectrum utilizing PET scan and rsfMRI. Dement. Geriatr. Cogn. Disord. Extra 11, 78–90. 10.1159/000516164 34178011 PMC8216015

[B26] Hercberg S. Chat-Yung S. Chauliac M. (2008). The French National Nutrition and Health Program: 2001–2006-?2010. Int. J. Public Health 53, 68–77. 10.1007/s00038-008-7016-2 18681335

[B27] Hohenfeld C. Werner C. J. Reetz K. (2018). Resting-state connectivity in neurodegenerative disorders: is there potential for an imaging biomarker? Neuroimage Clin. 18, 849–870. 10.1016/j.nicl.2018.03.013 29876270 PMC5988031

[B28] Janssen O. Jansen W. J. Vos S. J. Boada M. Parnetti L. Gabryelewicz T.. (2021). Characteristics of subjective cognitive decline associated with amyloid positivity. Alzheimers Dement. 18, 1832–1845. 10.1002/alz.12512 34877782 PMC9786747

[B29] Jenkinson M. Beckmann C. F. Behrens T. E. Woolrich M. W. Smith S. M. (2012). FSL. Neuroimage 62, 782–790. 10.1016/j.neuroimage.2011.09.015 21979382

[B30] Jensen C. S. Simonsen A. H. Siersma V. Beyer N. Frederiksen K. S. Gottrup H.. (2019). Patients with Alzheimer's disease who carry the APOE *E*4 allele benefit more from physical exercise. Alzheimers Dement. 5, 99–106. 10.1016/j.trci.2019.02.007 31011620 PMC6461575

[B31] Jernerén F. Cederholm T. Refsum H. Smith A. D. Turner C. Palmblad J.. (2019). Homocysteine status modifies the treatment effect of omega-3 fatty acids on cognition in a randomized clinical trial in mild to moderate Alzheimer's disease: the OmegAD study. J. Alzheimers. Dis. 69, 189–197. 10.3233/JAD-181148 30958356

[B32] Jockwitz C. Caspers S. (2021). Resting-state networks in the course of aging—differential insights from studies across the lifespan vs. amongst the old. Pflugers Arch Eur J Physiol 473, 793–803. 10.1007/s00424-021-02520-7 33576851 PMC8076139

[B33] Joffre C. Dinel A.-L. Chataigner M. Pallet V. Layé S. (2020). N-3 Polyunsaturated fatty acids and their derivates reduce neuroinflammation during aging. Nutrients 12, 647. 10.3390/nu12030647 32121189 PMC7146513

[B34] Kivipelto M. Mangialasche F. Ngandu T. (2018). Lifestyle interventions to prevent cognitive impairment, dementia and Alzheimer disease. Nat. Rev. Neurol. 14, 653–666. 10.1038/s41582-018-0070-3 30291317

[B35] Kivipelto M. Mangialasche F. Snyder H. M. Allegri R. Andrieu S. Arai H.. (2020). World-Wide FINGERS Network: a global approach to risk reduction and prevention of dementia. Alzheimers Dement. 16, 1078–1094. 10.1002/alz.12123 32627328 PMC9527644

[B36] Kivipelto M. Ngandu T. Laatikainen T. Winblad B. Soininen H. Tuomilehto J.. (2006). Risk score for the prediction of dementia risk in 20 years among middle aged people: a longitudinal, population-based study. Lancet Neurol. 5, 735–741. 10.1016/S1474-4422(06)70537-3 16914401

[B37] Lin C. Ly M. Karim H. T. Wei W. Snitz B. E. Klunk W. E.. (2020). The effect of amyloid deposition on longitudinal resting-state functional connectivity in cognitively normal older adults. Alzheimers Res. Ther. 12, 7. 10.1186/s13195-019-0573-1 31907079 PMC6945413

[B38] Marti del Moral A. Fortique F. (2019). Omega-3 fatty acids and cognitive decline: a systematic review. Nutr Hosp. 36, 939–949. 10.20960/nh.02496 31215788

[B39] Mora I. Arola L. Caimari A. Escoté X. Puiggròs F. (2022). Structured long-chain omega-3 fatty acids for improvement of cognitive function during aging. Int. J. Mol. Sci. 23, 3472. 10.3390/ijms23073472 35408832 PMC8998232

[B40] Park S.-J. Lee D.-K. Kim B. Na K.-S. Lee C.-H. Son Y.-D.. (2020). The association between omega-3 fatty acid intake and human brain connectivity in middle-aged depressed women. Nutrients 12, 2191. 10.3390/nu12082191 32717913 PMC7468955

[B41] Pottala J. V. Yaffe K. Robinson J. G. Espeland M. A. Wallace R. Harris W. S.. (2014). Higher RBC EPA + DHA corresponds with larger total brain and hippocampal volumes: WHIMS-MRI Study. Neurology 82, 435–442. 10.1212/WNL.0000000000000080 24453077 PMC3917688

[B42] Power J. D. Barnes K. A. Snyder A. Z. Schlaggar B. L. Petersen S. E. (2012). Spurious but systematic correlations in functional connectivity MRI networks arise from subject motion. Neuroimage 59, 2142–2154. 10.1016/j.neuroimage.2011.10.018 22019881 PMC3254728

[B43] Prinelli F. Fratiglioni L. Kalpouzos G. Musicco M. Adorni F. Johansson I.. (2019). Specific nutrient patterns are associated with higher structural brain integrity in dementia-free older adults. Neuroimage 199, 281–288. 10.1016/j.neuroimage.2019.05.066 31154046

[B44] Raji C. A. Erickson K. I. Lopez O. L. Kuller L. H. Gach H. M. Thompson P. M.. (2014). Regular fish consumption and age-related brain gray matter loss. Am. J. Prev. Med. 47, 444–451. 10.1016/j.amepre.2014.05.037 25084680 PMC4171345

[B45] Rakesh G. Szabo S. T. Alexopoulos G. S. Zannas A. S. (2017). Strategies for dementia prevention: latest evidence and implications. Ther. Adv. Chronic Dis. 8, 121–136. 10.1177/2040622317712442 28815009 PMC5546647

[B46] Rodrigues B. Asamane E. A. Magalhães R. Sousa N. Thompson J. L. Santos N. C. (2020). The association of dietary patterns with cognition through the lens of neuroimaging-a systematic review. Ageing Res. Rev. 63, 101145. 10.1016/j.arr.2020.101145 32818651

[B47] Salzman T. Sarquis-Adamson Y. Son S. Montero-Odasso M. Fraser S. (2022). Associations of multidomain interventions with improvements in cognition in mild cognitive impairment: a systematic review and meta-analysis. JAMA Netw Open 5, e226744. 10.1001/jamanetworkopen.2022.6744 35503222 PMC9066287

[B48] Samaille T. Fillon L. Cuingnet R. Jouvent E. Chabriat H. Dormont D.. (2012). Contrast-based fully automatic segmentation of white matter hyperintensities: method and validation. PLoS ONE 7, e48953. 10.1371/journal.pone.0048953 23152828 PMC3495958

[B49] Samieri C. Maillard P. Crivello F. Proust-Lima C. Peuchant E. Helmer C.. (2012). Plasma long-chain omega-3 fatty acids and atrophy of the medial temporal lobe. Neurology 79, 642–650. 10.1212/WNL.0b013e318264e394 22855869

[B50] Sánchez-Juan P. Seshadri S. (2017). Dynamic measurements of β-amyloid accumulation: the early effect of APOE. Neurology 89, 986–987. 10.1212/WNL.0000000000004344 28794255

[B51] Sivera R. Capet N. Manera V. Fabre R. Lorenzi M. Delingette H.. (2020). Voxel-based assessments of treatment effects on longitudinal brain changes in the multidomain Alzheimer preventive trial cohort. Neurobiol. Aging 94, 50–59. 10.1016/j.neurobiolaging.2019.11.020 32574818

[B52] Solomon A. Stephen R. Altomare D. Carrera E. Frisoni G. B. Kulmala J.. (2021). Multidomain interventions: state-of-the-art and future directions for protocols to implement precision dementia risk reduction. A user manual for Brain Health Services—part 4 of 6. Alzheimers Res. Ther. 13, 171. 10.1186/s13195-021-00875-8 34635167 PMC8507202

[B53] Sperling R. A. Donohue M. C. Raman R. Sun C.-K. Yaari R. Holdridge K.. (2020). Association of factors with elevated amyloid burden in clinically normal older individuals. JAMA Neurol. 77, 735. 10.1001/jamaneurol.2020.0387 32250387 PMC7136861

[B54] Stephen R. Liu Y. Ngandu T. Antikainen R. Hulkkonen J. Koikkalainen J.. (2019). Brain volumes and cortical thickness on MRI in the Finnish Geriatric Intervention Study to prevent cognitive impairment and disability (FINGER). Alzheimers. Res. Ther. 11, 53. 10.1186/s13195-019-0506-z 31164160 PMC6549301

[B55] Talukdar T. Zamroziewicz M. K. Zwilling C. E. Barbey A. K. (2019). Nutrient biomarkers shape individual differences in functional brain connectivity: evidence from omega-3 PUFAs. Hum. Brain Mapp. 40, 1887–1897. 10.1002/hbm.24498 30556225 PMC6865610

[B56] Tan Z. S. Harris W. S. Beiser A. S. Au R. Himali J. J. Debette S.. (2012). Red blood cell omega-3 fatty acid levels and markers of accelerated brain aging. Neurology 78, 658–664. 10.1212/WNL.0b013e318249f6a9 22371413 PMC3286229

[B57] Urchs S. Dansereau C. Benhajali Y. Bellec P. (2015). Group multiscale functional template generated with BASC on the Cambridge sample. 10.6084/M9.FIGSHARE.1285615.V128560308

[B58] van Balkom T. D. van den Heuvel O. A. Berendse H. W. van der Werf Y. D. Vriend C. (2020). The effects of cognitive training on brain network activity and connectivity in aging and neurodegenerative diseases: a systematic review. Neuropsychol. Rev. 30, 267–286. 10.1007/s11065-020-09440-w 32529356 PMC7305076

[B59] van Dalen J. W. Moll van Charante E. P. Caan M. W. Scheltens P. Majoie C. B. Nederveen A. J.. (2017). Effect of long-term vascular care on progression of cerebrovascular lesions: magnetic resonance imaging substudy of the prediva trial (prevention of dementia by intensive vascular care). Stroke 48, 1842–1848. 10.1161/STROKEAHA.117.017207 28596452

[B60] Vellas B. Carrie I. Gillette-Guyonnet S. Touchon J. Dantoine T. Dartigues J. F.. (2014). MAPT Study: a multidomain approach for preventing Alzheimer's disease: design and baseline data. J. Prev. Alzheimers Dis. 1, 13–22. 10.14283/jpad.2014.34 26594639 PMC4652787

[B61] Vettore M. De Marco M. Pallucca C. Bendini M. Gallucci M. Venneri A. (2021). White-matter hyperintensity load and differences in resting-state network connectivity based on mild cognitive impairment subtype. Front. Aging Neurosci. 13, 737359. 10.3389/fnagi.2021.737359 34690743 PMC8529279

[B62] Virtanen J. K. Siscovick D. S. Lemaitre R. N. Longstreth W. T. Spiegelman D. Rimm E. B.. (2013). Circulating omega-3 polyunsaturated fatty acids and subclinical brain abnormalities on MRI in older adults: the cardiovascular health study. JAHA 2. 10.1161/JAHA.113.000305 24113325 PMC3835236

[B63] Viviano R. P. Damoiseaux J. S. (2020). Functional neuroimaging in subjective cognitive decline: current status and a research path forward. Alzheimers Res. Ther. 12, 23. 10.1186/s13195-020-00591-9 32151277 PMC7063727

[B64] Walhovd K. B. (2014). Blood markers of fatty acids and vitamin D, cardiovascular measures, body mass index, and physical activity relate to longitudinal cortical thinning in normal aging. Neurobiol. Aging 35, 1055–64. 10.1016/j.neurobiolaging.2013.11.011 24332985

[B65] Wang Z. Williams V. J. Stephens K. A. Kim C.-M. Bai L. Zhang M.. (2020). The effect of white matter signal abnormalities on default mode network connectivity in mild cognitive impairment. Hum. Brain Mapp. 41, 1237–1248. 10.1002/hbm.24871 31742814 PMC7267894

[B66] Whitfield-Gabrieli S. Nieto-Castanon A. (2012). Conn: a functional connectivity toolbox for correlated and anticorrelated brain networks. Brain Connect. 2, 125–141. 10.1089/brain.2012.0073 22642651

[B67] Winkler A. M. Ridgway G. R. Webster M. A. Smith S. M. Nichols T. E. (2014). Permutation inference for the general linear model. Neuroimage 92, 381–397. 10.1016/j.neuroimage.2014.01.060 24530839 PMC4010955

[B68] Witte A. V. Kerti L. Hermannstädter H. M. Fiebach J. B. Schreiber S. J. Schuchardt J. P.. (2014). Long-chain omega-3 fatty acids improve brain function and structure in older adults. Cereb. Cortex 24, 3059–3068. 10.1093/cercor/bht163 23796946

[B69] Yao S. Liu Y. Zheng X. Zhang Y. Cui S. Tang C.. (2020). Do nonpharmacological interventions prevent cognitive decline? a systematic review and meta-analysis. Transl. Psychiatry 10, 19. 10.1038/s41398-020-0690-4 32066716 PMC7026127

[B70] Yurko-Mauro K. Alexander D. D. Van Elswyk M. E. (2015). Docosahexaenoic acid and adult memory: a systematic review and meta-analysis. PLoS ONE 10, e0120391. 10.1371/journal.pone.0120391 25786262 PMC4364972

[B71] Zhang Y. Chen J. Qiu J. Li Y. Wang J. Jiao J.. (2015). Intakes of fish and polyunsaturated fatty acids and mild-to-severe cognitive impairment risks: a dose-response meta-analysis of 21 cohort studies 1–3. Am. J. Clin. Nutr. 103, 330–340. 10.3945/ajcn.115.124081 26718417

[B72] Zwilling C. E. Talukdar T. Zamroziewicz M. K. Barbey A. K. (2019). Nutrient biomarker patterns, cognitive function, and fMRI measures of network efficiency in the aging brain. Neuroimage 188, 239–251. 10.1016/j.neuroimage.2018.12.007 30529508

