# Supplementary material for: Impact of multidomain preventive strategies on functional brain connectivity in older adults with cognitive complaint: Subset from the Montpellier center of the ancillary MAPT-MRI study
Source: Front Aging Neurosci. 2023 Jan 10;14:971220. doi: 10.3389/fnagi.2022.971220 (PMC9871772; doi:10.3389/fnagi.2022.971220)
Supplement: Supplementary file 1 [file Data_Sheet_1.docx]

**SUPPLEMENTARY MATERIALS**

**Table S1 : Characteristics of the subgroups of interest from the MAPT trial which were not examined in our analyses due to limited group sizes.**

|  | **MRI subsample (N=100)** | **Omega 3+MI**  **(N=27)** | **Omega3**  **(N=24)** | **MI (N=24)** | **Placebo**  **(N=25)** | **p*** |
| --- | --- | --- | --- | --- | --- | --- |
| **APOE e4 allele** |  |  |  |  |  | 0.409 |
| Carriers | 23 (25%) | 7 (28%) | 4 (18%) | 8 (36%) | 4 (17%) |  |
| Non carriers | 69 (75%) | 18 (72%) | 18 (82%) | 14 (64%) | 19 (83%) |  |
| **MMSE score at baseline** |  |  |  |  |  | 0.961 |
| MMSE < 30 | 87 (87%) | 24 (89%) | 21 (88%) | 21 (88%) | 21 (84%) |  |
| MMSE = 30 | 13 (13%) | 3 (11%) | 3 (12%) | 3 (12%) | 4 (16%) |  |
| **At risk for dementia** |  |  |  |  |  | 0.046 |
| Low dementia risk (CAIDE score < 6) | 16 (16%) | 4 (15%) | 7 (29%) | 0 (0%) | 5 (20%) |  |
| High dementia risk (CAIDE score >= 6) | 84 (84%) | 23 (85%) | 17 (71%) | 24 (100%) | 20 (80%) |  |
| **DHA and EPA in red blood cells** |  |  |  |  |  | 0.318 |
| Low DHA and EPA in red blood cells | 15 (16%) | 4 (17%) | 5 (21%) | 5 (22%) | 1 (4%) |  |
| Normal DHA and EPA in red blood cells | 80 (84%) | 20 (83%) | 19 (79%) | 18 (78%) | 23 (96%) |  |
| **SUV** |  |  |  |  |  | 0.353 |
| SUV positive | 14 (47%) | 1 (17%) | 4 (44%) | 3 (60%) | 6 (60%) |  |
| SUV negative | 16 (53%) | 5 (83%) | 5 (56%) | 2 (40%) | 4 (40%) |  |

*Comparison between intervention groups. Kruskall-Wallis and Anova or Chi-2 tests were used for quantitative and qualitative variables respectively.

Percentages were calculated with the number of participants for whom data was available for each variable. Percentages were rounded to the nearest value. This rounding can result in a loss of accuracy and the sum of the percentages may be close but not equal to 100%.

Abbreviations : APOE=apolipoprotein; MMSE=Mini Mental State Examination; CAIDE=Cardiovascular Risk Factors, Aging, and Incidence of Dementia; DHA=docosahexaenoic acid; EPA=eicosapentaenoic acid; SUV=standardized uptake value.

**Table S2 : Characteristics and comparisons of included and excluded subjects.**

|  | Included subjects (N=100) | Excluded subjects  (N=88) | p* |
| --- | --- | --- | --- |
| **Age, years (mean, (sd) / range)** | 74.26 (3.74 [70.00 ; 84.00]) | 74.49 (3.78 [70.00 ; 84.00]) | 0.329 |
| **Sex** |  |  | 0.578 |
| Men | 37 (37%) | 37 (42%) |  |
| Women | 63 (63%) | 51 (58%) |  |
| **Education Level** |  |  | 0.251 |
| < University level | 51 (51%) | 52 (60%) |  |
| ≥ University level | 49 (49%) | 34 (40%) |  |
| **Composite score (mean, (sd) / range)** |  |  |  |
| At baseline | 0.16 (0.51 [-1.60 ; 1.27]) | 0.05 (0.59 [-1.59 ; 1.48]) | 0.158 |
| Difference from baseline to 36 month | 0.14 (0.49 [-1.61 ; 1.11]) | -0.01 (0.48 [-1.83 ; 1.04]) | **0.011** |
| **MMSE score (mean, (sd) / range)** |  |  |  |
| At baseline | 28.01 (1.38 [24.00 - 30.00]) | 28.25 (1.57 [24.00 - 30.00]) | 0.060 |
| Difference from baseline to 36 month | 0.44 (1.73 [-5.00 ; 6.00]) | 0.08 (1.68 [-5.00 ; 5.00]) | **0.057** |
| **CDR status at baseline** |  |  | 0.839 |
| CDR0 | 52 (52%) | 48.0 (55%) |  |
| CDR0.5 | 48 (48%) | 40 (45%) |  |
| **CDR status evolution from baseline to 36 months** |  |  | 0.595 |
| CDR0 to CDR0 | 36 (36%) | 28 (32%) |  |
| CDR0.5 to CDR0.5 | 25 (25%) | 22 (25%) |  |
| CDR0 to CDR0.5 | 16 (16%) | 20 (23%) |  |
| CDR0.5 to CDR0 | 23 (23%) | 17 (19%) |  |
| CDR0.5 to CDR1 | 0 (0%) | 1 (0%) |  |

*Comparison between included and excluded subjects groups. Mann-Whitney and two-sample t test or Chi-2 tests were used for quantitative and qualitative variables respectively. Percentages were calculated with the number of participants for whom data was available for each variable. Percentages were rounded to the nearest value. This rounding can result in a loss of accuracy and the sum of the percentages may be close but not equal to 100%.

Abbreviations : MMSE=Mini Mental State Examination; CDR=clinical dementia rating.

**Table S3 : Significant connections over time for interaction between intervention group and baseline CDR in the unadjusted model. Results presented are significant after SPC FWE-cluster correction. Connections in bold remain significant after adjustement for age, sex and level of education.**

| Cluster statistics | Individual connection statistics | Subnetwork (Main network) 1 | Subnetwork (Main network) 2 | Mean connectivity per intervention group within CDR0 subgroup | Mean connectivity per intervention group within CDR0.5 subgroup |
| --- | --- | --- | --- | --- | --- |
| p-unc < 0.001  p-FWE=0.007 | F=6.57  p < 0.001 | **L/R Cun/SOG (VIS)** | **L/R MFG/MSFG (FPN)** | Om3+MI= -0.01  Om3= -0.04  MI= 0.19  Pl= 0.20 | Om3+MI= 0.13  Om3= 0.11  MI= -0.21  Pl= -0.06 |
| - | F=5.94  p=0.001 | **L/R SMG/Post CG (SN)** | **L/R MFG/SMG (FPN)** | Om3+MI= -0.03  Om3= -0.07  MI= 0.21  Pl= 0.24 | Om3+MI= 0.05  Om3= 0.09  MI= -0.07  Pl= -0.09 |
| - | F=5.01  p=0.003 | **L/R Pre CG (SMN)** | **L/R MFG/SMG (FPN)** | Om3+MI= -0.01  Om3= -0.01  MI= 0.16  Pl= 0.27 | Om3+MI= 0.13  Om3= -0.01  MI= -0.05  Pl= -0.05 |
| - | F=5.29  p=0.002 | **L/R Sup Pre/Post CG (SMN)** | **L/R MFG/MSFG (FPN)** | Om3+MI= 0.07  Om3= -0.02  MI= 0.18  Pl= 0.30 | Om3+MI= 0.17  Om3= 0.02  MI= -0.15  Pl= -0.13 |
| - | F=5.10  p=0.003 | **L/R Sup Pre/Post CG (SMN)** | **L/R MFG/SMG (FPN)** | Om3+MI= -0.00  Om3= -0.01  MI= 0.20  Pl= 0.25 | Om3+MI= 0.13  Om3= 0.01  MI= -0.03  Pl= -0.07 |
| - | F=4.56  p=0.005 | **L/R Pre/Post CG (SMN)** | **L/R MFG/SMG (FPN)** | Om3+MI= 0.08  Om3= -0.05  MI= 0.21  Pl= 0.33 | Om3+MI= 0.16  Om3= 0.03  MI= -0.05  Pl= 0.02 |
| - | F=4.65  p=0.005 | **L/R SPL (SN)** | **L/R MFG/SMG (FPN)** | Om3+MI= -0.02  Om3= -0.06  MI= 0.11  Pl= 0.16 | Om3+MI= 0.09  Om3= -0.06  MI= -0.16  Pl= -0.12 |
| - | F=4.60  p=0.005 | **L/R SMG/Post CG (SN)** | **L/R MFG/MSFG (FPN)** | Om3+MI= 0.06  Om3= -0.02  MI= 0.14  Pl= 0.21 | Om3+MI= 0.03  Om3= 0.20  MI= -0.07  Pl= -0.13 |
| p-unc=0.007  p-FWE=0.049 | F=7.27  p < 0.001 | L/R SPL/MOG (FPN) | L/R SFG (SN) | Om3+MI= -0.13  Om3= -0.03  MI= -0.17  Pl= 0.28 | Om3+MI= 0.05  Om3= -0.08  MI= 0.06  Pl= -0.03 |
| - | F=6.15  p < 0.001 | L/R MTG/ITG (FPN) | L/R MCgG (SN) | Om3+MI= -0.11  Om3= -0.09  MI= 0.04  Pl= 0.19 | Om3+MI= 0.03  Om3= -0.01  MI= -0.04  Pl= -0.20 |
| - | F=4.99  p=0.003 | L/R SPL/MOG (FPN) | L/R AIns (SN) | Om3+MI= 0.05  Om3= -0.02  MI= 0.05  Pl= 0.24 | Om3+MI= 0.16  Om3= 0.12  MI= -0.14  Pl= -0.12 |
| - | F=4.74  p=0.004 | L/R SPL/MOG (FPN) | L/R MCgG (SN) | Om3+MI= -0.05  Om3= -0.03  MI= -0.14  Pl= 0.22 | Om3+MI= 0.06  Om3= 0.04  MI= 0.07  Pl= -0.04 |

Abbreviations : VIS=Visual network; FPN=Frontoparietal network; SN=Salience network; SMN=Sensorimotor network; L/R Cun/SOG=Left/Right Cuneus/Superior Occipital Gyrus; L/R MFG/MSFG=Left/Right Middle Frontal Gyrus/Superior Frontal Gyrus Medial segment; L/R SMG/Post CG=Left/Right Supramarginal Gyrus/Post Central Gyrus; L/R MFG/SMG=Left/Right Middle Frontal Gyrus/Supramarginal Gyrus; L/R Pre CG=Left/Right Pre Central Gyrus; L/R Sup Pre/Post CG=Left/Right Superior Pre/Post Central Gyrus; L/R Pre/Post CG=Left/Right Pre/Post Central Gyrus;L/R SPL=Left/Right Superior Parietal Lobule; L/R SPL/MOG=Left/Right Superior Parietal Lobule/Middle Occipital Gyrus; L/R SFG=Left/Right Frontal Gyrus; L/R MTG/ITG=Left/Right Middle Temporal Gyrus/Inferior Temporal Gyrus; L/R MCgG=Left/Right Middle Cingulate Gyrus; L/R AIns=Left/Right Anterior Insula; SPC : Spatial Pairwise Clustering; FWE : Family-Wise Error.

**Figure S1 : Mean raw evolution of connectivity over 36 months for connections differing on participants CDR status and intervention groups (Table 2).**


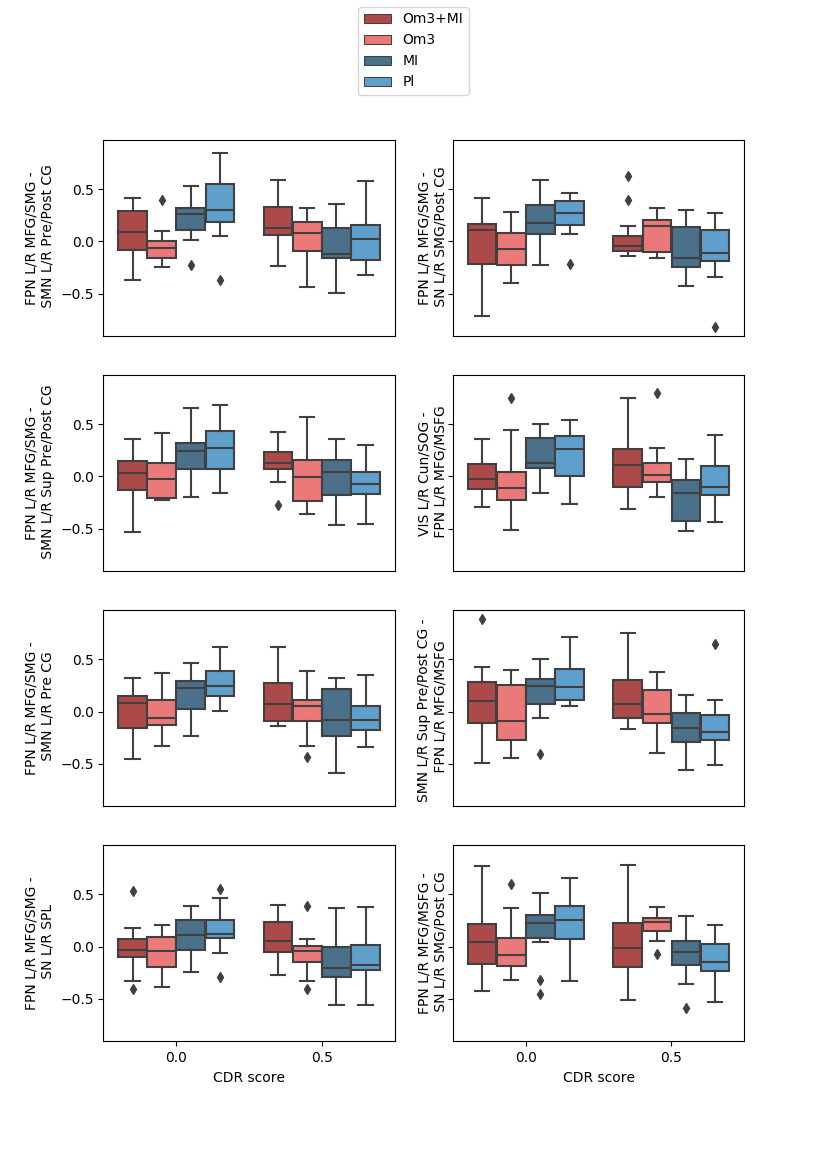


Abbreviations : FPN=Frontoparietal network; SMN=Sensorimotor network; SN=Salience network; VIS=Visual network; L/R Cun/SOG=Left/Right Cuneus/Superior Occipital Gyrus; L/R MFG/MSFG= Left/Right Middle Frontal Gyrus/Superior Frontal Gyrus Medial segment; L/R SMG/Post CG= Left/Right Supramarginal Gyrus/Post Central Gyrus; L/R MFG/SMG=Left/Right Middle Frontal Gyrus/ Supramarginal Gyrus; L/R Pre CG=Left/Right Pre Central Gyrus; L/R Sup Pre/Post CG=Left/Right Superior Pre/Post Central Gyrus; L/R Pre/Post CG=Left/Right Pre/Post Central Gyrus; L/R SPL=Left/Right Superior Parietal Lobule

**Table S4 : Atlas subnetworks’ abbreviations and supplementary information.**

| Subnetwork | Abbreviation subnetwork | Network* | Atlas index** | MNI coordinates*** |
| --- | --- | --- | --- | --- |
| Left/Right Middle Frontal Gyrus/Supramarginal Gyrus | L/R MFG SMG | FPN (FPN=98%, SN=2%) | 35 | x=-45 y=19 z=22  x=44 y=25 z=20  x=-36 y=-58 z=52  x=44 y=-46 z=52 |
| Left/Right Pre/Post Central Gyrus | L/R Pre/Post CG | SMN (100%) | 3 | x=-57 y=-6 z=29  x=56 y=-4 z=28 |
| Left/Right Superior Pre/Post Central Gyrus | L/R Sup Pre/Post CG | SMN (100%) | 9 | x=-42 y=-22 z=55  x=42 y=-16 z=55 |
| Left/Right Supramarginal Gyrus/Post Central Gyrus | L/R SMG/Post CG | SN (SN=72%, SMN=28%) | 33 | x=-50 y=-32 z=43  x=48 y=-29 z=42 |
| Left/Right Pre Central Gyrus | L/R Pre CG | SMN (100%) | 15 | x=-16 y=-25 z=65  x=16 y=-25 z=65 |
| Left/Right Cuneus/Superior Occipital Gyrus | L/R Cun/SOG | VIS (100%) | 11 | x=-15 y=-88 z=35  x=15 y=-88 z=35 |
| Left/Right Middle Frontal Gyrus/Superior Frontal Gyrus Medial segment | L/R MFG/MSFG | FPN (FPN=68%,  SN=24%,  DMN=8%) | 29 | x=-31 y=44 z=23  x=27 y=47 z=23  x=-6 y=27 z=37  x=6 y=27 z=37 |
| Left/Right Superior Parietal Lobule | L/R SPL | SN (SN=53%, SMN=47%) | 27 | x=-21 y=-49 z=67  x=21 y=-49 z=67 |
| Left/Right Inferior Occipital Gyrus/Middle Occipital Gyrus | L/R IOG/MOG | VIS (100%) | 24 | x=-38 y=-81 z=3  x=36 y=-71 z=0 |
| Left/Right Lingual Gyrus | L/R LiG | VIS (100%) | 26 | x=-12 y=-59 z=4  x=12 y=-59 z=4 |
| Left/Right Anterior Insula | L/R AIns | SN (100%) | 28 | x=-42 y=14 z=1  x=43 y=16 z=3 |
| Left/Right Putamen | L/R Put | SN (100%) | 2 | x=-24 y=2 z=1  x=24 y=2 z=1 |
| Left/Right Middle Cingulate Gyrus | L/R MCgG | SN (SN=85%, SMN=15%) | 14 | x=-11 y=-24 z=41  x=11 y=-24 z=41 |
| Left/Right Superior Parietal Lobule/Middle Occipital Gyrus | L/R SPL/MOG | FPN (100%) | 22 | x=33 y=-67 z=40  x=-27 y=-73 z=34 |
| Left/Right Frontal Gyrus | L/R SFG | SN (SN=98%, SMN=2%) | 21 | x=-28 y=0 z=57 |
| Left/Right Middle Temporal Gyrus/Inferior Temporal Gyrus | L/R MTG/ITG | FPN (100%) | 16 | x=-51 y=-52 z=-8  x=51 y=-52 z=-8 |
| Right Middle Frontal Gyrus/Angular Gyrus | R MFG/AG | FPN (FPN=93%, DMN=7%) | 23 | x=51 y=-52 z=40  x=33 y=26 z=43 |
| Left/Right Superior Frontal Gyrus / Left Angular Gyrus | L/R SFG L AG | DMN (DMN=74%, FPN=26%) | 36 | x=-19 y=41 z=45  x=19 y=41 z=45  x=-50 y=-58 z=38 |
| Left/Right Middle Frontal Gyrus/triangular part of the inferior frontal gyrus | L/R MFG/TrIFG | FPN (FPN=70%, DMN=30%) | 34 | x=-45 y=44 z=-2  x=36 y=44 z=-8 |

* Subnetwork network’s affiliation was determined by selecting the R7 atlas network which overlapped the most with the subnetwork. Percentages of overlap between an R36 subnetwork and R7 networks are reported.

** Index corresponds to subnetwork index in the atlas.

*** Coordinates in mm of the different regions in the Montreal Neurological Institute coordinate system.

**Table S5 : Significant connections after SPC FWE-cluster correction for interaction over time between omega-3 intake ((Om3 & Om3 + MI) vs (MI & Placebo)) and baseline CDR status, and subsequent post hoc analysis within subgroups of participants CDR0 and CDR0.5. All significant connections are part of the same cluster.**

|  | **SPC individual connections statistics for interaction omega-3 intake x CDR status x time (t ; pvalue *)** | **CDR0 at baseline (t ; pvalue *)**  **Omega-3 intake (N=31) ≠ No Omega-3 intake (N=21)** | | **CDR0.5 at baseline (t ; pvalue *)**  **Omega-3 intake (N=20) ≠ No Omega-3 intake (N=28)** | |
| --- | --- | --- | --- | --- | --- |
| **Connections from subnetwork L/R MFG/SMG (FPN) to subnetworks** |  | Omega 3 > no Omega3 | Mean connectivity | Omega 3 > no Omega3 | Mean connectivity |
| L/R SMG/Post CG (SN) | T=-4.19  p < 0.001 | **T=-3.95**  **p < 0.001** | Om3=-0.05  No Om3=0.22 | T=1.85  p=0.071 | Om3=0.06  No Om3=-0.08 |
| L/R SPL (SN) | T= -3.86  p < 0.001 | **T=-2.85**  **p=0.007** | Om3=-0.04  No Om3=0.13 | **T=2.65**  **p=0.011** | Om3=0.03  No Om3=-0.14 |
| L/R Pre CG (SMN) | T = -3.85 p < 0.001 | **T=-3.88**  **p < 0.001** | Om3=-0.01  No Om3=0.21 | T=1.90  p=0.064 | Om3=0.07  No Om3=-0.05 |
| L/R Sup Pre/Post CG (SMN) | T=-3.86 p < 0.001 | **T=-3.71**  **p < 0.001** | Om3=-0.01  No Om3=0.23 | T=1.56  p=0.127 | Om3=0.08  No Om3=-0.05 |
| L/R Pre/Post CG (SMN) | T=-3.86 p < 0.001 | **T=-3.62**  **p < 0.001** | Om3=0.02  No Om3=0.27 | **T=2.03**  **p=0.049** | Om3=0.10  No Om3=-0.01 |
| **Connections from subnetwork**  **L/R MFG/MSFG (FPN) to subnetworks** |  |  |  |  |  |
| L/R Cun/SOG (VIS) | T=-4.29 p<0.001 | **T=-2.98**  **p=0.005** | Om3=-0.02  No Om3=0.20 | **T=2.97**  **p=0.005** | Om3=0.13  No Om3=-0.13 |
| L/R Sup Pre/Post CG (SMN | T -4.00 p < 0.001 | **T=-2.68**  **p=0.010** | Om3=0.021  No Om3=0.23 | **T=3.02**  **p=0.004** | Om3=0.11  No Om3=-0.13 |
| L/R SMG/Post CG (SN) | T=-3.23 p=0.002 | **T=-2.05**  **p=0.046** | Om3=0.02  No Om3=0.17 | **T=2.42**  **p=0.020** | Om3=0.10  No Om3=-0.10 |
| L/R Pre CG (SMN) | T =-2.75 p=0.007 | T=-1.72  p=0.091 | Om3=0.07  No Om3=0.23 | **T=2.19**  **p=0.034** | Om3=0.12  No Om3=-0.06 |
| L/R Pre/Post CG (SMN) | T =-3.13 p=0.002 | **T=-2.62**  **p=0.012** | Om3=-0.01  No Om3=0.20 | T=1.90  p=0.064 | Om3=0.07  No Om3=-0.03 |
| **Connections from source R MFG/AG (FPN)** |  |  |  |  |  |
| L/R Cun/SOG (VIS) | T=-3.21 p=0.002 | **T=-2.09**  **p=0.042** | Om3=-0.01  No Om3=0.21 | **T=2.77**  **p=0.008** | Om3=0.17  No Om3=-0.02 |
| L/R Sup Pre/Post CG (SMN) | T=-4.38 p < 0.001 | **T=-3.75**  **p < 0.001** | Om3=-0.02  No Om3=0.27 | **T=2.26**  **p=0.029** | Om3=0.14  No Om3=-0.03 |
| L/R Pre/Post CG (SMN) | T=-4.08  p < 0.001 | **T=-3.83**  **p < 0.001** | Om3=-0.01  No Om3=0.26 | **T=2.11**  **p=0.040** | Om3=0.11  No Om3=-0.02 |
| L/R SMG/Post CG (SN) | T=-2.75 p=0.007 | **T=-2.97**  **p=0.005** | Om3=0.00  No Om3=0.24 | T=0.79  p=0.434 | Om3=0.05  No Om3=-0.02 |
| L/R Pre CG (SMN) | T=-2.93 p=0.004 | **T=-3.46**  **p=0.001** | Om3=-0.02  No Om3=0.28 | T=0.65  p=0.517 | Om3=0.06  No Om3=0.03 |
| L/R SPL (SN) | T=-3.28 p=0.001 | **T=-2.67**  **p=0.010** | Om3=-0.03  No Om3=0.19 | **T=2.09**  **p=0.043** | Om3=0.03  No Om3=-0.09 |
| **Connections from source L/R SFG L AG (DMN)** |  |  |  |  |  |
| L/R Cun/SOG (VIS) | T=-2.67 p=0.009 | T=-1.23  p=0.226 | Om3=-0.03  No Om3=0.10 | **T=2.57**  **p=0.014** | Om3=0.17  No Om3=-0.06 |
| L/R Sup Pre/Post CG (SMN) | T=-2.90 p=0.005 | T=-1.81  p=0.076 | Om3=0.02  No Om3=0.14 | **T=2.16**  **p=0.036** | Om3=0.14  No Om3=-0.05 |
| **Connections from source L/R MFG/TrIFG(FPN)** |  |  |  |  |  |
| L/R Pre/Post CG (SMN) | T=-2.64 p=0.01 | T=-1.37  p=0.178 | Om3=0.10  No Om3=0.21 | **T=2.59**  **p=0.013** | Om3=0.13  No Om3=-0.03 |

Significant results are outlined in bold; *The model was adjusted for age, sex and level of education; Abbreviations : VIS=Visual network; FPN=Frontoparietal network; SN=Salience network; SMN=Sensorimotor network; DMN=Default Mode Network; L/R MFG/SMG=Left/Right Middle Frontal Gyrus/Supramarginal Gyrus; L/R SMG/Post CG=Left/Right Supramarginal Gyrus/Post Central Gyrus; L/R SP=Left/Right Superior Parietal Lobule;L/R Pre CG=Left/Right Pre Central Gyrus;L/R Sup Pre/Post CG=Left/Right Superior Pre/Post Central Gyrus; L/R Pre/Post CG=Left/Right Pre/Post Central Gyrus; L/R MFG/MSFG=Left/Right Middle Frontal Gyrus/Superior Frontal Gyrus Medial segment; L/R Cun/SOG=Left/Right Cuneus/Superior Occipital Gyrus; R MFG/AG=Right Middle Frontal Gyrus/Angular Gyrus; L/R SFG L AG=Left/Right Superior Frontal Gyrus Left Angular Gyrus; L/R MFG/TrIFG=Left/Right Middle Frontal Gyrus/triangular part of the inferior frontal gyrus.

**Table S6 : Connection tending to be significant after SPC FWE-cluster correction for stable CDR0 participants when contrasting omega-3 intake (Om3 & Om3 + MI, N=20) > no-omega-3 (MI & Placebo, N=16) intake.**

| Cluster statistics | Individual connection statistics | Subnetwork (Main network) 1 | Subnetwork (Main network) 2 |
| --- | --- | --- | --- |
| p-unc=0.009  p-FWE=0.067 | T=-3.93  p < 0.001 | L/R SMG/Post CG (SN) | L/R MFG/SMG (FPN) |
|  | T=-3.57  p=0.001 | L/R Pre CG (SMN) | L/R MFG/SMG (FPN) |
|  | T=-3.16  p=0.003 | L/R Pre/Post CG (SMN) | L/R MFG/SMG (FPN) |
|  | T=-2.96  p=0.006 | L/R Sup Pre/Post CG (SMN) | L/R MFG/SMG (FPN) |

Abbreviations : FPN=Frontoparietal network; SN=Salience network; SMN=Sensorimotor network; L/R SMG/Post CG=Left/Right Supramarginal Gyrus/Post Central Gyrus; L/R MFG/SMG=Left/Right Middle Frontal Gyrus/Supramarginal Gyrus; L/R Pre CG =Left/Right Pre Central Gyrus; L/R Sup Pre/Post CG=Left/Right Superior Pre/Post Central Gyrus; L/R Pre/Post CG= Left/Right Pre/Post Central Gyrus; SPC=Spatial Pairwise Clustering; FWE=Family-Wise Error.

**Table S7 : Connection tending to be significant after SPC FWE-cluster correction for stable CDR0.5 participants when contrasting omega-3 intake (Om3 & Om3 + MI, N=10) > no-omega-3 (MI & Placebo, N=15) intake.**

| Cluster statistics | Individual connection statistics | Subnetwork (Main network) 1 | Subnetwork (Main network) 2 |
| --- | --- | --- | --- |
| p-unc=0.011  p-FWE=0.078 | T=3.87  p<0.001 | L/R LiG (VIS) | L/R AIns (SN) |
|  | T=3.39  p=0.003 | L/R IOG/MOG (VIS) | L/R AIns (SN) |
|  | T=3.30  p=0.004 | L/R LiG (VIS) | L/R Put (SN) |
|  | T=3.20  p=0.005 | L/R IOG/MOG (VIS) | L/R Put (SN) |

Abbreviations : VIS=Visual network; SN=Salience network;L/R LiG=Left/Right Lingual Gyrus; L/R AIns=Left/Right Anterior Insula; L/R IOG/MOG=Left/Right Inferior Occipital Gyrus/Middle Occipital Gyrus; L/R Put=Left/Right Putamen; SPC=Spatial Pairwise Clustering; FWE=Family-Wise Error.
